# Plasma Exosomes at the Late Phase of Remote Ischemic Pre-conditioning Attenuate Myocardial Ischemia-Reperfusion Injury Through Transferring miR-126a-3p

**DOI:** 10.3389/fcvm.2021.736226

**Published:** 2021-11-30

**Authors:** Danni Li, Yang Zhao, Chuyi Zhang, Fan Wang, Yan Zhou, Sanqing Jin

**Affiliations:** Department of Anesthesia, The Sixth Affiliated Hospital, Sun Yat-sen University, Guangzhou, China

**Keywords:** remote ischemic pre-conditioning, exosomes, microRNA, miR-126a-3p, cardioprotection, myocardial ischemia-reperfusion, cardiomyocyte apoptosis

## Abstract

**Background:** Remote ischemic pre-conditioning (RIPC) alleviated the myocardial ischemia-reperfusion injury, yet the underlying mechanisms remain to be fully elucidated, especially at the late phase. Searching a key component as a transfer carrier may provide a novel insight into RIPC-mediated cardioprotection in the condition of myocardial ischemia-reperfusion.

**Objective:** To investigate the cardioprotective effect of plasma exosomes at the late phase of RIPC and its potential signaling pathways involved.

**Methods and Results:** Exosomes were isolated from the plasma of rats 48 h after the RIPC or control protocol. Although the total plasma exosomes level had no significant change at the late phase of RIPC (RIPC-exosome) compared with the control exosomes (Control-exosome), the RIPC-exosome afforded remarkable protection against myocardial ischemia-reperfusion (MI/R) injury in rats and hypoxia-reoxygenation (H/R) injury in cells. The miRNA array revealed significant enrichment of miR-126a-3p in RIPC-exosome. Importantly, both miR-126a-3p inhibitor and antagonist significantly blunted the cardioprotection of RIPC-exosome in H/R cells and MI/R rats, respectively, while miR-126a-3p mimic and agomir showed significant cardioprotection against H/R injury in cells and MI/R injury in rats. Mechanistically, RIPC-exosome, especially exosomal miR-126a-3p, activated the reperfusion injury salvage kinase (RISK) pathway by enhancing the phosphorylation of Akt and Erk1/2, and simultaneously inhibited Caspase-3 mediated apoptotic signaling.

**Conclusions:** Our findings reveal a novel myocardial protective mechanism that plasma exosomes at the late phase of RIPC attenuate myocardial ischemia-reperfusion injury via exosomal miR-126a-3p.

## Introduction

It has been well-known that remote ischemic pre-conditioning (RIPC), a phenomenon where transient non-fatal ischemia of distant organs from the heart, protects the myocardium against myocardial ischemia-reperfusion (MI/R) injury (1). The protective effects of RIPC have early phase and late phase (2, 3). The early phase protection which occurs immediately after RIPC lasts for about 3 h. The late phase protection which appears 24–48 h after RIPC, lasts for 3–4 days and has lots of biology efficacies (4). The mechanisms underlying the cardioprotective effects of RIPC, especially at the late phase, remain elusive.

Many studies support that blood-borne mediators serve as signal transduction mechanisms (5, 6). Several studies have focused on exploring the nature of circulatory mediators in the blood that may carry pre-conditioning signals from remote organs to target organs, binds to respective receptors, and activates intracellular signaling pathways (7–13). Our previous studies show the cardioprotection of RIPC at the late phase can be transferred by plasma (14–17), but many questions about the mechanisms remain unanswered.

Exosomes are small (50–90 nm) endogenous membrane vesicles secreted by a variety of cells and are thought to be good mediators of intercellular communication and crosstalk through the transfer of various signaling molecules, including proteins, mRNAs, and non-coding RNAs such as miRNAs (18). In the last decade, the functions of exosomes in MI/R injury have been widely explored (19–23). It has been reported that long-term exercise-derived exosomes have cardioprotection against MI/R injury with exosomal miR-342-5p as a novel cardioprotective exerkine (24). Vincencio et al. proposed that exosomes induced by the RIPC at the early phase may act as carriers of the cardioprotective factors (25). Minghua et al. found that exosomal miR-24 at the early phase of RIPC in rats may play a central role in mediating the cardioprotective effects of RIPC (26). These findings suggest that exosomes may play a role in the cardiovascular benefits conferred by RIPC. Since small amounts of exosomes may have significant effects (27), it is crucial to explore the functional effects of exosomes, especially their cardioprotection in the late phase of RIPC.

In this study, we found that the cardioprotective effect could be transferred by plasma exosomes at the late phase of RIPC and exosomal miR-126a-3p might be a novel cardioprotective molecule against MI/R injury, which activated the RISK pathway and inhibited the activation of apoptotic protein Caspase-3.

## Materials and Methods

### Animals

Male Sprague-Dawley rats (250–300 g, aged 7–8 weeks) were obtained from the Experimental Animal Center of Southern Medical University (China) and housed in separate cages in a temperature-controlled room (22–24°C) with free access to water and food. We used male rats in this study to avoid the possible interference of estrogen hormonal disturbances on the results because studies have shown that estrogen *per se* exerts cardiovascular protective effects. Rats were randomly assigned to either experimental or a control group, using a completely randomized design. This study was approved by the Animal Care Ethics Committees of the Sixth Affiliated Hospital, Sun Yat-sen University (IACUC-20190221-002 and IACUC-2020091101). Rats were anesthetized with an intraperitoneal injection of 3% sodium pentobarbital (60 mg/kg).

### Animal Model of RIPC

RIPC was operated by binding elastic rubber bands around the proximal ends of both hind limbs with a 4-cycle procedure (5 min ischemia followed by 5 min reperfusion) after the rats were anesthetized. Cessation of blood flow to the hind limbs was confirmed using laser Doppler imaging to monitor microcirculatory blood flow in the hind limbs (Laser Doppler Blood Perfusion Imager, Perimed AB, Sweden). After 48 h, the rats in both groups were anesthetized, placed in a supine position, laparotomy performed, blood was drawn from the abdominal aorta using a 7^#^ needle and rapidly removed into a vacutainer containing sodium citrate.

### Isolation of Exosomes and Free-Exosomes Plasma

Exosomes were prepared by differential centrifugation at 4°C as follows. Whole blood samples were centrifuged at 1,600 × g for 20 min to obtain plasma, then centrifuged at 10,000 × g for 45 min to remove platelets and cells, and then twice at 100,000 × g for 70 min with a Type 100 Ti rotor (Beckman, Germany). After a series of differential centrifugation steps above, the final precipitation was considered as exosomes. Exosomes isolated from 1 ml of plasma were redissolved in 100 μl of PBS and filtered by a 0.22 μm pore filter and stored at −80°C for further experiments. *In vivo* study, 100 μL of the exosome suspension was further diluted to 1 ml before use. *In vitro* study, 100 μl of the exosome suspension was added to each well.

Free-exosome plasma was prepared by differential centrifugation at 4°C as follows. Whole blood samples were centrifuged at 1,600 xg for 20 min to obtain supernatant, then centrifuged at 2,000 × g for 45 min and 10,000 × g for 45 min to remove cells and platelets, and then at 110,000 × g for 70 min with a Type 100 Ti rotor. After that, the supernatant was considered as free-exosome plasma. Free-exosome plasma was also filtered using a 0.22 μm pore filter and stored at −80°C.

### Transmission Electron Microscopy (TEM)

Transmission electron microscopy (FEI, Thermo Fisher, USA) was used to observe the exosomes. Exosomes purified from 1,000 μL of plasma were resuspended in 1,000 μl of PBS. Then, 10 μL of the exosome solution was loaded on a formvar/carbon-coated 200-mesh copper electron microscopy grid and incubated for 2 min at 25°C, and then was stained with 3% phosphotungstic acid for 2 min at 25°C. After that, the exosomes were visualized using a transmission electron microscope. Micrographs were used to quantify the diameter of exosomes.

### Nanoparticle Tracking Analysis

Nanoparticle tracking analysis (NTA) was used to analyze the absolute size distribution of exosomes given that the Brownian movement of nanoparticles in a solution is related to their size (28). Exosomes isolated from 1 ml of plasma were resuspended in 1 ml of PBS,1:1000, dilutions were analyzed in NanoSight NS300 (Marvel, UK). The following settings were used: measurement temperature of 20 ± 1°C, 30 frames per second, and measurement time of 60 s. Each sample was tested thrice.

### Cell Culture

The rat cardio-myoblast cell line (H9C2) was purchased from the Cell Bank of the Chinese Academy of Sciences. The H9C2 cells were cultured in a complete medium containing Dulbecco's modified Eagle's medium (DMEM) (Gibco, Thermo Fisher, USA), 10% fetal bovine serum (Gibco, Thermo Fisher, USA), and 1% penicillin-streptomycin (Sigma-Aldrich, USA).

Neonatal rat primary cardiomyocytes were isolated from 1or 2 days old Sprague Dawley rats. The ventricular parts of the hearts were minced into 1–2 mm^3^ tissue blocks under sterile conditions. The tissues were repeatedly digested with freshly prepared compound digestive enzyme (collagenase II 1 mg/ml, 0.2 mg/ml in D-Hank's solution, sterile filtered, pH 7.2. Gibco, Thermo Fisher, USA) for 20 min in at 37°C until they were completely digested and then filtered through a 200 μm cell strainer. Filtered cardiac cells were cultured in a plate and incubated at 37°C for 1 h to remove fibroblasts. The suspensions were centrifuged at 1,000 rpm for 5 min at 4°C and then the cardiomyocytes were cultured in DMEM/F12 (Gibco, Thermo Fisher, USA) medium supplemented with 10% fetal bovine serum, 1% penicillin/streptomycin at 37°C in humid air with 5% CO_2_ for 36 h before other treatments.

To study the functions of exosomes, exosomes purified from 1 ml of plasma and resuspended in 100 ul of PBS were cultured with cells in a 6-well-plate for 24 h before the induction of Hypoxia/Reoxygenation injury.

### Exosome Labeling

To confirm exosome uptake by H9C2 cells, exosomes were labeled using a Dio green fluorescence kit (MCE, USA) according to the manufacturer's protocol. Lipophilic tracers Dio were prepared in stock solutions of dimethyl sulfoxide (DMSO) for the *in vitro* study. Exosomes were stained with 10 μM Dio for 10 min at 4°C and ultracentrifuged twice at 100,000 *g* for 40 min at 4°C to remove the excess dye. The isolated exosomes were resuspended in DMEM and used in the uptake experiments. Cells in the experimental group were incubated with Dio-stained exosomes and cells in the blank group were incubated only with Dio without exosomes. After a 2 h incubation, the cells were fixed with 4% paraformaldehyde (TCI, Japan) for 30 min and then visualized using a fluorescence microscope (IX73, Olympus, Japan) with 4′,6-diamidino-2-phenylindole, dihydrochloride (DAPI) stain. Dio emits green fluorescence under the excitation of light with a wavelength of 484 nm.

### Cell Model of Hypoxia/Reoxygenation

The cells in serum-free and glucose-free DMEM were placed in a hypoxic chamber containing 95% N_2_/5% CO_2_ for 24 h. Subsequently, cells were subjected to reoxygenation in a standard incubator (95% air/5% CO_2_) in a complete medium for 6 h.

### Cell Viability Assay

Cell viability was observed using a Cell Counting kit-8 (CCK-8; Ape×bio, USA) according to the manufacturer's instructions. Cells were cultured into 96-well-plates at a density of 2,000 cells per well in a complete culture medium. Five replicates were set up for each group. The cells were treated according to the grouping. Subsequently, 10 μl CCK-8 was added to each well and incubated for 2 h. Optical density values were measured at 450 nm on a microplate reader (Multiskan FC, Thermo Fisher, USA).

### Flow Cytometry

Fluorescein isothiocyanate (FITC)-conjugated Annexin V and propidium iodide (PI) (BD Biosciences, USA) were used to identify apoptotic cells. The left upper quadrant (FITC–/PI+) showed the dead cells; the right upper quadrant (FITC+/PI+) showed the late apoptotic cells; the left lower quadrant (FITC–/PI–) showed the intact cells, and the right lower quadrant (FITC+/PI–) showed the early apoptotic cells. Experiments were performed using a flow cytometer (Beckman, Germany), and the data obtained from the cell population were analyzed with Flow Jo_V10 (BD Biosciences, USA) software. The rate of total apoptotic cells was counted as the sum of early and late apoptotic rates.

### Animal Model of Myocardial Ischemia/Reperfusion

Rats were anesthetized and intubated for mechanical ventilation in a supine position. The respiratory rate was 70 cycles/min, the tidal volume was 1 ml per 100 g body weight and the inspiration and expiration ratio was 1: 1 (Double channel Rodent Ventilator, ZH-DW-3000S, Zhenhua, China). A heating pad was used to keep rats' body temperature at around 37°C. Hemodynamic indices were recorded with a multi-channel physiological signal acquisition processing system (PowerLab, AD Instruments) that was connected to a pressure transducer. Then, the chest of a rat was opened via a lateral thoracotomy, and the heart was exposed through a pericardiotomy. A 6–0 nylon suture with a curved tapered needle was placed under the left anterior descending coronary artery (LAD) 3 mm below the left atrium. A small plastic tube was placed above the myocardium to reduce direct dissecting injury to the myocardium. The surgeon ligated the suture line with the small plastic tube for reversible LAD occlusion. Myocardial ischemia was confirmed by visual myocardium cyanosis and S-T segment elevation on electrocardiography. After 30 min LAD-ligation and the small plastic tube remove, reperfusion began and continued for 180 min. Finally, the rats were sacrificed after the reperfusion for histologic assessment. All procedures and analyses were performed by investigators blinded to the grouping. Rats that died from complications during anesthesia and surgery were excluded from the analysis. The sample size was chosen based on our previous experience in the study of animal models of myocardial ischemia/reperfusion.

### Tail Vein Injection of Exosomes or Free-Exosomes Plasma

Tail vein injection of exosomes or free-exosomes plasma was performed on rats 24 h before the induction of MI/R injury as described above. Briefly, male Sprague-Dawley rats (6 weeks old) were placed in a fixator. Exosomes purified from 1 ml of plasma and resuspended in 1 ml of PBS, or 1 ml of free-exosomes plasma were injected into the tail vein. The experiments were blindly operated by a researcher who did not know the grouping.

### Echocardiography Measurements

Functional cardiac changes before the intervention and after the reperfusion were determined using echocardiography. The operator was blinded to the treatment. Transthoracic two-dimensional M-mode echocardiography was performed using the Vevo 2100 Imaging System (Visual Sonics, USA) equipped with an MS-250 probe (29–31). Left ventricular end-systolic and end-diastolic diameters (LVSD and LVDD, respectively), were measured after obtaining a 2-dimensional short-axis view of the left ventricle at the level of the papillary muscles. The mean value of the three measurements was determined for each sample. Left ventricular end-diastolic volume (EDV) and end-systolic volume (ESV) were calculated according to the Teichholz formula. The left ventricular ejection fraction (LVEF) was calculated as (EDV–ESV)/EDV × 100% (32). Changes in cardiac function were determined by the difference in the LVEF (ΔLVEF).

### Measurement of Myocardial Infarct Size

Myocardial infarct size was determined by the Evans blue/triphenyl tetrazolium chloride (TTC) double staining method. After 180 min reperfusion, the LAD was ligated again. Two min later, the rat was injected with 2 ml of 3% Evans blue (Sigma-Aldrich, USA) via the femoral vein. The area of heart unstained by Evans blue was named the area at risk (AAR). After death from an overdose of sodium pentobarbital, the heart was immediately removed and washed off with 0.9% saline. Subsequently, the heart was frozen at −20°C for 60 min and then cut into 5 equal thickness transverse slices (2 mm each) from the apex to the base. These slices were placed in 1% TTC (TCI, Japan) for 10 min in the dark at 37°C, then fixed in 4% paraformaldehyde (TCI, Japan) for 24 h. After TTC staining, the white area was known as the infarct area (INF) and the red area was the viable myocardium in the AAR. Images were taken and quantified by planimetry with Image J software (version 1.52a, National Institutes of Health, USA) in a blinded manner. Myocardial infarct size was represented as the percentage of INF relative to the AAR (INF/AAR × 100%).

### TUNEL Assay for Myocardial Apoptosis

Myocardial apoptosis was expressed by terminal deoxynucleotidyl transferase-mediated dUTP nick-end labeling (TUNEL) staining. An *In Situ* Cell Death Detection Kit (Roche, USA) was used according to the manufacturer's instructions. Briefly, the anterior wall of the left ventricular myocardium was cut into the freezing section. Apoptotic nuclei in the sections appeared green stained with the TUNEL, whereas all nuclei appeared blue by DAPI (4, 6-diamino-2-phenylindole; Beyotime, China). Each section was photographed at 400-magnification with a fluorescence microscope (IX73, Olympus, Japan). The apoptotic index was represented as the percentage of apoptotic myocytes relative to the total number of cardiomyocytes (green/blue) using Image J software (version 1.52a) in a blinded manner.

### The miRNA Library Construction and Sequencing

Total RNAs from plasma exosomes isolated from the control rats and the RIPC rats were extracted using the Trizol reagent (Invitrogen, Carlsbad, CA, USA) according to the manufacturer's instructions (*n* = 3 per group). The miRNA library preparation and sequencing were conducted using an Illumina HiSeq 2500 platform (Ribobio, China). Both 3' and 5' adaptors were ligated with unique small RNA fractions to construct a small RNA sequencing library. Subsequently, the adaptor-ligated RNA fragments were reverse-transcribed and amplified using PCR and sequenced. Differential expression of miRNAs between the two groups was analyzed using cluster analysis.

### Transfection of Cells

The miR-126a-3p mimics (50 nmol/L), inhibitors (100 nmol/L), and their negative control (50/100 nmol/L) RNAs (RiboBio, China) were transfected into cells with Lipofectamine 3000 (Life Technologies, USA) according to the manufacturer's instructions. Cells were incubated for 6 h with the transfection complex before it was discarded and replaced by fresh media. Experiments were performed 24 h after transfection.

### Intracardiac Injection of miRNA Agomir or Antagomir

The miR-126a-3p agomir, antagomir, and their negative control (agomir-NC, antagomir-NC) (RiboBio, China) were used according to the manufacturer's instructions. Rats were intracardiac injected with 10 nmol miR-126a-3p antagomir, or 5 nmol miR-126a-3p agomir, or their negative control in 200 μl of the saline buffer. Briefly, anesthetized male Sprague-Dawley rats were placed in a supine position with respiratory support by a respiratory mask during the surgery, and the fourth intercostal space was exposed without thoracotomy or cutting the ribs. The 30-gauge needle was inserted at the place where the heart apical pulse was strongest. The needle tip was determined to be located in the heart cavity when pulsating blood return was sawn which was consistent with the heart pulse frequency. The chest wall was then closed following the injection. After 24 h, these rats were subjected to MI/R injury as described above in a blinded manner.

### RNA Isolation and Quantitative Real Time-PCR

Exosome-RNAs were isolated from exosomes with Trizol (Invitrogen, Carlsbad, CA, USA) according to the standard procedures and cel-miR-39 (RiboBio, China) was added to normalize the technical variation between samples. Tissue and cell RNAs were extracted with RNA-Quick Purification Kit for small RNA (ES Science, China) according to the protocols obtained from the manufacturers, and U6 (RiboBio, China) was used as the internal reference for miRNA. RNA concentrations were verified on the NanoDrop Spectrophotometer (Thermo Scientific, USA). Isolated RNA was reversely transcribed using miRNA primers (Bulge-LoopTM miRNA RT primer) and the riboSCRIPT Reverse Transcription Kit (RiboBio, China). qRT-PCR was performed on a Roche LightCycler 480°C System (Roche, Switzerland) using a TB Green Premix Ex Taq II Kit (Takara, Japan). Amplification was performed at 95°C for 30 s, followed by 40 cycles of 95°C for 5 s and 60°C for 20 s. All procedures were performed according to the protocols obtained from the manufacturers. Fold change in RNA species was calculated using formula 2^(−ΔΔ^Ct). ΔΔCt = (Ct target miRNA–Ct control). The sequences were as follows:

miR-126a-3p 5′-UCGUACCGUGAGUAAUAAUGCG-3′

let-7c-5p 5′-UGAGGUAGUAGGUUGUAUGGUU-3′

let-7f-5p 5′-UGAGGUAGUAGAUUGUAUAGUU-3′

miR-203a-3p 5′-GUGAAAUGUUUAGGACCACUAG-3′

miR-144-3p 5′-UACAGUAUAGAUGAUGUACU-3′

miR-98-5p 5′-UGAGGUAGUAAGUUGUAUUGUU-3′.

### Western Blot

Tissues, cells, and purified exosomes were lysed with RIPA buffer (Beyotime, China). Protein concentration was determined using a BCA Protein Assay kit (Beyotime, China). Protein samples were separated using electrophoresis with SDS-PAGE (sodium dodecyl sulfate-polyacrylamide gel electrophoresis) and transferred onto a polyvinylidene difluoride (PVDF) membrane (Roche Diagnostics, USA). The membranes were blocked with 5% skimmed milk in TBS-T (Tris-buffered saline with 0.1% Tween 20) and incubated with the appropriate primary antibodies at 4°C for 12 h, followed by incubation with the corresponding secondary antibodies at 25°C for 1 h. The blots were detected by a Bio-Rad (California, USA) automatic chemiluminescence imaging analysis system using ECL supersensitive luminescent solution (Thermo Fisher, USA). TBS-T was used to wash the membranes. Primary antibodies against CD9 (ab92726), CD81 (ab109201) were purchased from Abcam (USA) and p-Akt (Ser473) (#4060), Akt (#2920), p-Erk1/2 (Thr202/Tyr204) (#4370), Erk1/2 (#4695), Caspase-3 (#14220), cleaved Caspase-3 (#9661), LY294002 (#9901), U0126 (#9903), and GAPDH (#97166) were purchased from Cell Signaling Technology (CST, USA). Secondary antibody goat anti-mouse IgG (ab205719) and goat anti-rabbit IgG (ab6721) were purchased from Abcam (USA). Antibodies against GAPDH were used as loading controls.

### Statistical Analysis

All of the statistical tests and were performed with the SPSS 26.0 software (SPSS Inc., Chicago, IL, USA). Statistical significance was considered as *p* < 0.05. All values are presented as mean ± standard deviations of *n* independent experiments. Normality of data distribution was assessed using the Shapiro-Wilk normality test and the homogeneity of variance was tested by the Leven test. If the data of two groups conformed to normal distribution and homogeneity of variance, they were analyzed by two-tailed Student *t*-test, otherwise, they were analyzed by Mann-Whitney *U*-test. If other data conformed to normal distribution and homogeneity of variance, they were analyzed by ANOVA (three or more groups) followed by Bonferroni's correction, otherwise, they were analyzed by Kruskal-Wallis test.

## Results

### Identification and Functional Verification of Plasma Exosomes at the Late Phase of RIPC *in vitro*

**Figure 1:**
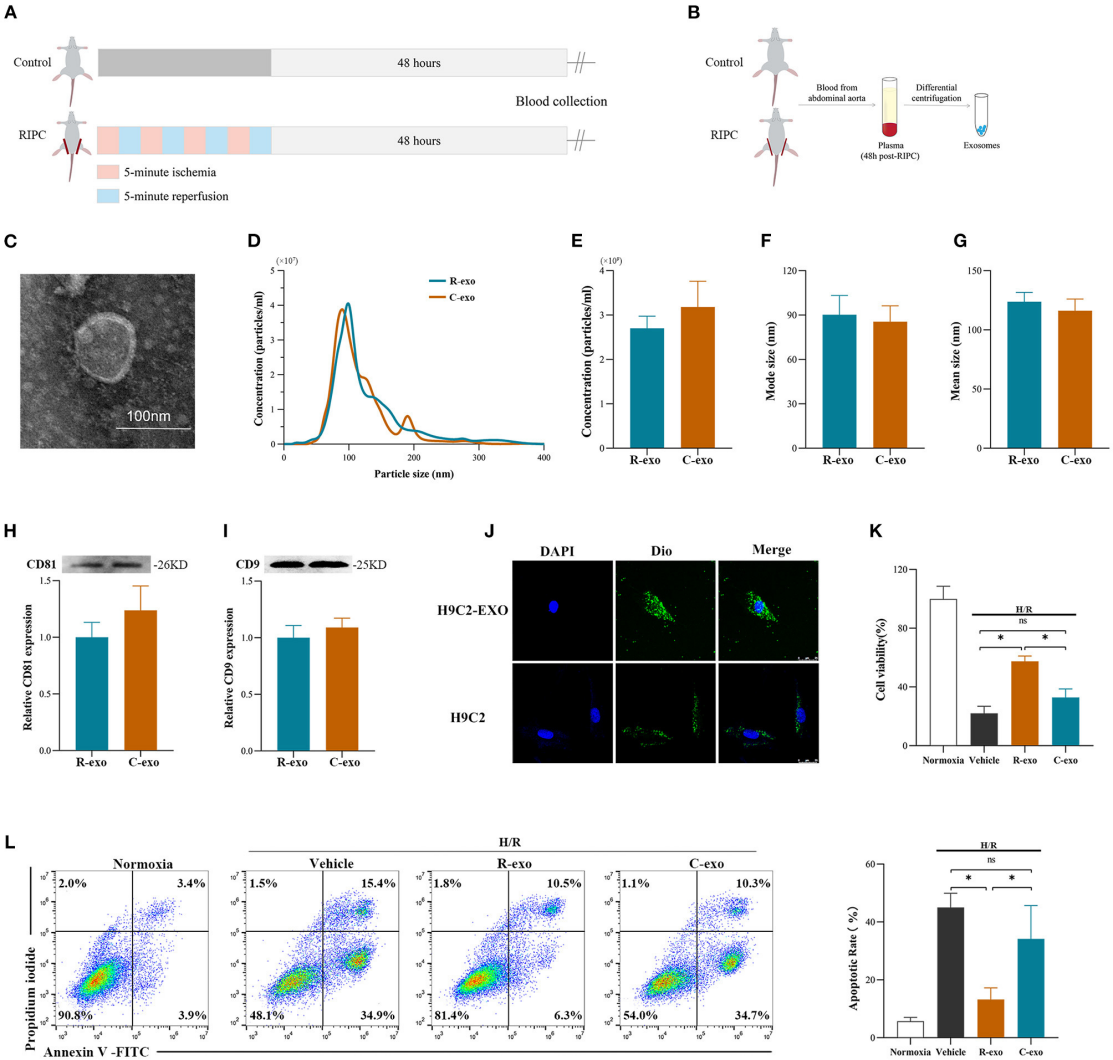
Identification and functional verification of plasma exosomes at the late phase of RIPC *in vitro*. **(A)** Establishment of the late-phase RIPC model in rats. **(B)** The process to obtain the plasma exosomes from rats with or without the late-phase RIPC protocol. **(C)** Representative electron micrograph of isolated plasma exosomes. Scale bar: 100 nm. **(D)** Representative results of NTA (nanoparticle tracking analysis) demonstrating size distribution and concentration in Control-exosomes (C-exo) and RIPC-exosomes (R-exo). **(E)** The average concentration in Control-exosomes and RIPC-exosomes (*n* = 4). **(F)** Mode size in Control-exosomes and RIPC-exosomes (*n* = 4). **(G)** Mean size in Control-exosomes and RIPC-exosomes (*n* = 4). **(H)** Representative images of Western blot (top) and quantified data (bottom) in exosomal marker proteins CD81 in Control-exosomes and RIPC-exosomes (*n* = 4). **(I)** Representative images of Western blot (top) and quantified data (bottom) in exosomal marker proteins CD9 in Control-exosomes and RIPC-exosomes (*n* = 4). **(J)** Representative images of H9C2 cells that were incubated with the presence (H9C2-EXO) or absence (H9C2) of Dio-labeled exosomes (green). Cardiomyocyte nuclei were stained with DAPI (blue). Scale bar: 25 μm. **(K)** The data of cell viability showed that RIPC-exosomes improved cell viability with 24 h co-incubation and following H/R injury. **(L)** The data and representative images of cell apoptosis by flow cytometric analysis showed that RIPC-exosomes decreased cell apoptosis with 24 h co-incubation and following H/R injury (*n* = 3). RIPC, remote ischemic pre-conditioning; R-exo, exosomes isolated from the plasma of the late-phase RIPC rats; C-exo, exosomes isolated from the plasma of the control rats; H/R, Hypoxia/Reoxygenation (24/6 h). *p < 0.05 and ^ns^*p* > 0.05.

To better understand the function of plasma exosomes mediated cardioprotection induced by the late phase of RIPC, plasma exosomes were isolated from control rats (Control-exosome, C-exo) and RIPC-rats (RIPC-exosome, R-exo) 48 h after the RIPC protocol using the traditional differential ultracentrifugation (Figures 1A,B). Transmission electron microscopy revealed typical circular particles with diameters of 50–90 nm in separated sections (Figure 1C). Nanoparticle tracking analysis represented no significant differences in size distribution and concentration (2.7 × 10^9^ ± 0.3 × 10^9^ ml^−1^ vs. 3.2 × 10^9^ ± 0.6 × 10^9^ ml^−1^) between Control-exosome and RIPC-exosome (Figures 1D–G). Western blot analysis confirmed the presence and the similar levels of exosome marker proteins (CD81 and CD9) between RIPC-exosome and Control-exosome (Figures 1H,I). These results confirmed that exosomes had been successfully isolated and purified and the total plasma exosomes level had no significant change between RIPC-exosome and Control-exosome.

To confirm whether exosomes could be internalized by H9c2 cells, we incubated the H9C2 cells with Dio-labeled exosomes and observed that exosomes were taken up by H9C2 cells as indicated by confocal images (Figure 1J). Subsequently, to demonstrate the protective effects of RIPC-exosome, we performed functional experiments and the study design was shown in Supplementary Figure 2A. Importantly, pre-incubation with RIPC-exosome, but not Control-exosome, for 24 h attenuated cellular H/R injury as indicated by increased cell viability (Figure 1K) and reduced cell apoptosis (Figure 1L) when compared with the vehicle control. Collectively, these results showed that RIPC-exosome conferred protective effects against H/R injury in H9C2 cells.

### Plasma Exosomes at the Late Phase of RIPC Exerted Cardioprotection Against MI/R Injury *in vivo*

**Figure 2:**
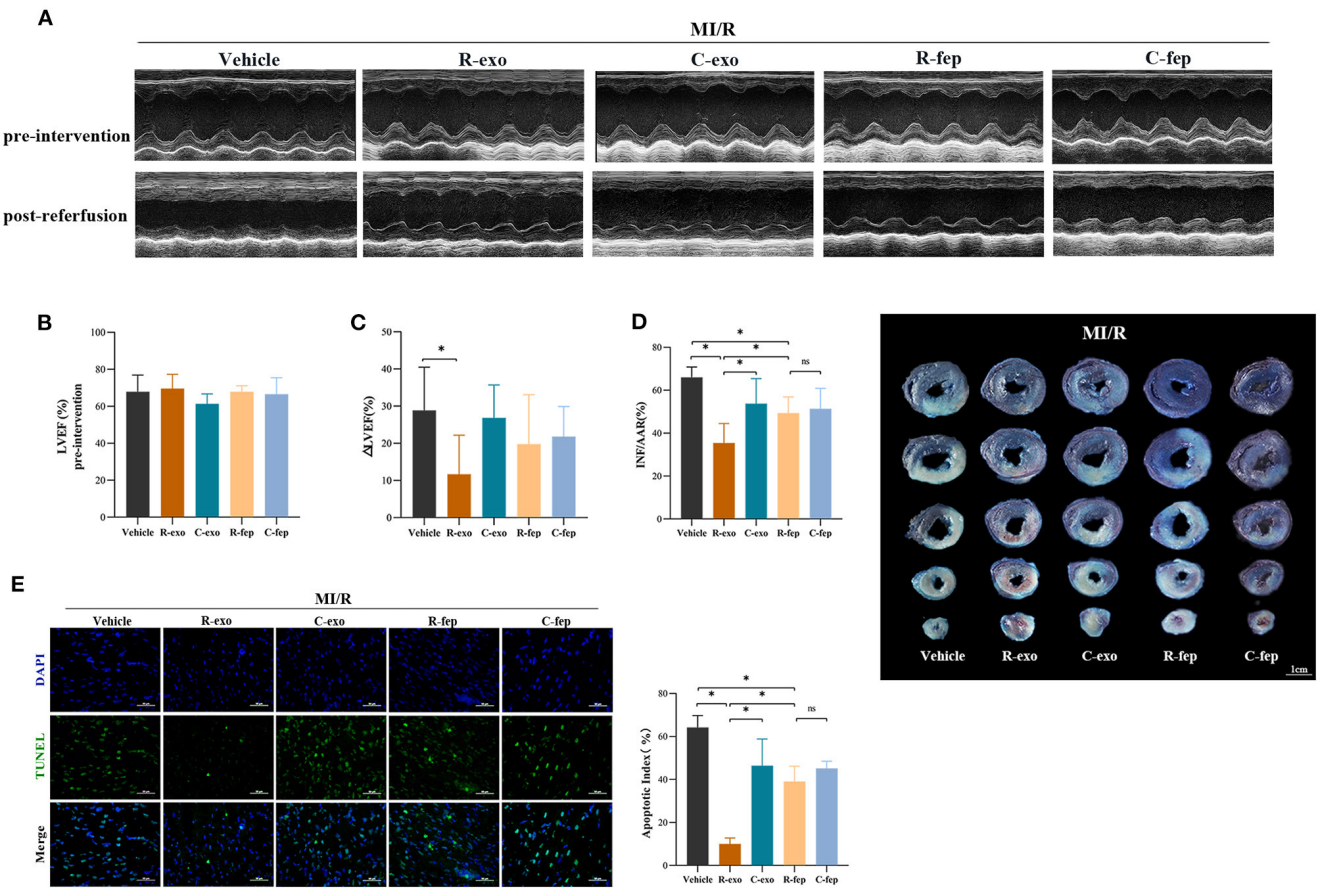
Plasma exosomes at the late phase of RIPC exerted cardioprotection against MI/R injury *in vivo*. **(A)** Representative echocardiographic images of rats before the intervention (pre-intervention) and after the reperfusion (post-reperfusion) in each group of rats. **(B)** Left ventricular ejection fraction (LVEF) of rats in pre-intervention (*n* = 8). **(C)** ΔLVEF (the difference between the LVEF in pre-intervention and that in post-reperfusion) in each group. RIPC-exosome injected into rats for 24 h before MI/R improved cardiac function as evidence by significantly narrowed the ΔLVEF. **(D)** Quantified data and presentative photographs of heart sections. RIPC-exosome decreased infarct size in rats subjected to MI/R (*n* = 8). INF/AAR, infarct size as a percentage of myocardial area at risk. **(E)** Representative images and quantified data of TUNEL assay in each group showed that RIPC-exosome decreased myocardial apoptosis in rats subjected to MI/R. Myocardial apoptosis in rats determined by a percent of TUNEL-positive nuclei (green)/total nuclei (blue) (*n* = 4). Scale bar: 50 μm. MI/R, myocardial ischemia/reperfusion (30/180 min). R-exo, exosomes isolated from the plasma of the late-phase RIPC rats. C-exo, exosomes isolated from the plasma of the control rats. R-fep, the supernatant collected after the first ultracentrifugation from the plasma of the late-phase RIPC rats. C-fep, the supernatant collected after the first ultracentrifugation from the plasma of the control rats. *p < 0.05, ^ns^*p* > 0.05.

To determine the cardioprotection of RIPC-exosome *in vivo*, exosomes or free-exosomes plasma were injected into rats 24 h before MI/R. This *in vivo* study design was shown in Supplementary Figure 2B. RIPC-exosome (R-exo) or Control-exosome (C-exo) purified from 1 ml of plasma were resuspended in 1 ml of PBS (≈2.9 × 10^9^ exosomes). The injection volume of free-exosomes plasma, isolated from the RIPC-rats (R-fep) or the control-rats (C-fep), was 1 ml. The cardiac function was assessed by the difference in LVEF before the intervention and after the reperfusion (ΔLVEF%). The cardiac function after MI/R injury had significant improvement in the R-exo group compared with that in the vehicle group as evidenced by narrowed the ΔLVEF (11.7 ± 10.5% vs. 28.8 ± 11.7%, *p* < 0.05, Figures 2A–C). Concomitantly, the infarct size relative to the area at risk (INF/AAR) was significantly smaller in the R-exo group compared with that in the vehicle group after MI/R injury (35.5 ± 9.1% vs. 66.0 ± 4.8%, *p* < 0.05, Figure 2D). Besides, reduced cell apoptosis was observed in the R-exo group compared with that in the vehicle group following MI/R (10.0 ± 2.7% vs. 64.2 ± 5.6%, *p* < 0.05; Figure 2E).

Taken together, these *in vivo* data suggested that transfer of RIPC-exosome improved cardiac function, reduced infarct size, and cell apoptosis in MI/R-induced rats.

### miR-126a-3p Was Essential in the Cardioprotection Provided by Plasma Exosomes at the Late Phase of RIPC

**Figure 3:**
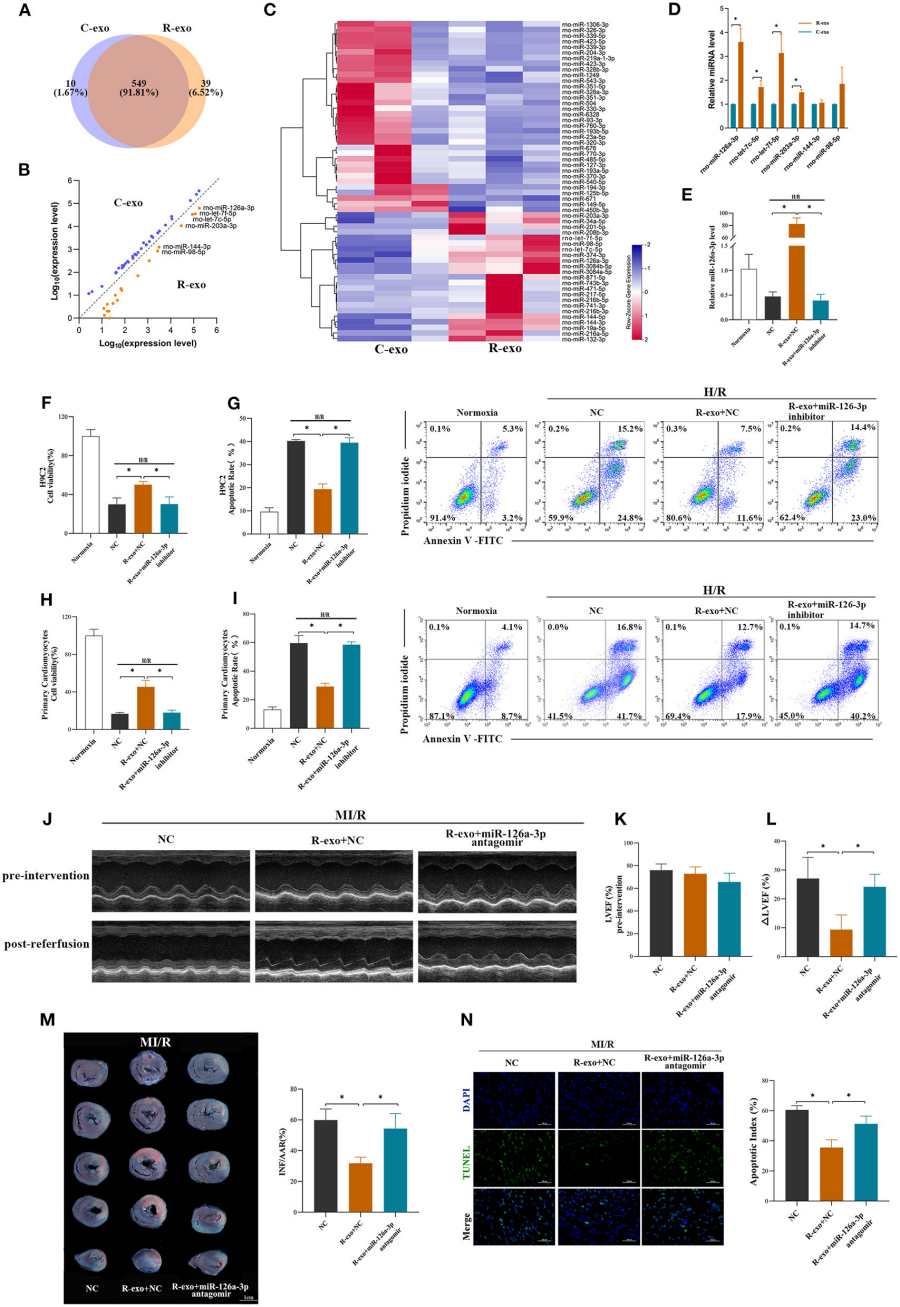
miR-126a-3p was essential in the cardioprotection provided by plasma exosomes at the late phase of RIPC. **(A)** Venn diagram showing the numbers of overlapping and unique miRNAs detected in Control-exosome (C-exo) and RIPC-exosome (R-exo) (*n* =3). **(B)** The expression level of miRNAs in Control-exosome and RIPC-exosome. Yellow dots represented up-regulated miRNAs in RIPC-exosome and blue dots represented up-regulated miRNAs in Control-exosome (fold change >2 and *p* < 0.05, *n* =3). **(C)** Heat map representing the miRNA profiling assays between Control-exosome and RIPC-exosome (fold change >2 and *p* < 0.05, *n* =3). **(D)** qRT-PCR analysis of the six differentially expressed miRNAs in Control-exosome and RIPC-exosome. Data are normalized to cel-miR-39 (*n* =3). **(E)** The qRT-PCR analysis of miR-126a-3p level in H9C2 cells treated with inhibitor negative control (NC), R-exo + NC, or R-exo + miR-126a-3p inhibitor followed by H/R. Data were presented as a fold change of the normoxia. U6 was used as an internal control (*n* = 3). **(F)** The data of cell viability showed that inhibition of miR-126a-3p attenuated the protective effects of RIPC-exosome in H9C2 cells subjected to H/R (*n* = 3). **(G)** The data and representative images of apoptosis of H9C2 cells subjected to H/R by flow cytometric analysis (*n* = 3). The protective effects of RIPC-exosome were eliminated by inhibition of miR-126a-3p. **(H)** The data of cell viability showed that the protective effects of RIPC-exosomal miR-126a-3p were reduced by a miR-126a-3p inhibitor in primary cardiomyocytes following H/R injury (*n* =3). **(I)** The data and representative images of apoptosis of primary cardiomyocytes subjected to H/R by flow cytometric analysis (*n* = 3). The anti-apoptosis effects of RIPC-exosomal miR-126a-3p were eliminated by inhibition of miR-126a-3p (*n* =3). **(J)** Representative echocardiographic images of rats in pre-intervention and post-reperfusion in each group of rats (*n* = 5). **(K)** Left ventricular ejection fraction (LVEF) of rats in pre-intervention. **(L)** ΔLVEF in each group showed that miR-126a-3p antagomir blunted the protective effects of RIPC-exosome in cardiac function. **(M)** Representative photographs of heart sections and quantified data demonstrated that miR-126a-3p antagomir observably attenuated the protective effects of RIPC-exosome in myocardial infarct size (*n* = 5). **(N)** Representative images and quantified data of TUNEL assay in each group demonstrated that miR-126a-3p antagomir observably blunted the protective effects of RIPC-exosome in myocardial apoptosis (*n* = 4). Scale bar: 50 μm. R-exo, exosomes isolated from the plasma of the late-phase RIPC rats. C-exo, exosomes isolated from the plasma of the control rats. H/R, Hypoxia/Reoxygenation (24/6 h). MI/R, myocardial ischemia/reperfusion (30/180 min). *p < 0.05.

It has been indicated that exosomal miRNAs may be an attractive candidate as a mediator of signal transmission in cardiovascular disease (18, 33, 34). To explore the cardioprotective effects of miRNAs induced by RIPC-exosome, we performed a miRNA profiling assay (763 rat miRNAs) comparing with RIPC-exosome (R-exo) and Control-exosome (C-exo) using Illumina HiSeq 2500 high-throughput sequencing. Overlapping miRNAs accounted for 91.81% of the total, which indicates that there was a minor change in the RIPC-induced exosomal miRNAs (Figure 3A). Subsequently, a total of 57 differentially expressed miRNAs were detected (fold change >2.0; *p* < 0.05), and 23 of them were highly expressed in RIPC-exosome compared with those in Control-exosome (Figures 3B,C). Moreover, 6 miRNAs of the 23 ones, which were highly expressed and closely related to myocardial apoptosis and protective effects such as the promotion of myocardial angiogenesis, were further confirmed by qRT-PCR analysis. On comparing RIPC-exosome with Control-exosome, miR-126a-3p was upregulated most significantly in RIPC-exosome (Figure 3D). To better understand the level of exosomal miR-126a-3p in hearts after tail vein injection of plasma exosomes and MI/R injury, we assessed the level of miR-126a-3p in the heart by qRT-PCR. The miR-126a-3p level was elevated in hearts injected with RIPC-exosome following the treatments (Supplementary Figure 3).

To explore the *in vitro* function of exosomal miR-126a-3p using the model of H/R injury, the study design was shown in Supplementary Figure 4A. We first assessed levels of miR-126a-3p in H9C2 cells treated with RIPC-exosome. The miR-126a-3p level was significantly elevated in H9C2 cells incubated with RIPC-exosome and this change was significantly blunted with the transfection with miR-126a-3p inhibitor (Figure 3E), indicating that RIPC-exosome delivered miR-126a-3p to H9C2 cells. Subsequently, in both H9C2 cells and primary cardiomyocytes, the protective improvement in cell viability and cell apoptosis exerted by RIPC-exosome were markedly eliminated by inhibition of miR-126a-3p (Figures 3F–I).

Moreover, to further understand the *in vivo* function of exosomal miR-126a-3p induced by RIPC, we injected miR-126a-3p antagomir into rats and established the model of MI/R in rats. The study design was shown in Supplementary Figure 4B. Briefly, our results show that the miR-126a-3p antagomir injection markedly attenuated the cardioprotective effects of the RIPC-exosome. Firstly, improved cardiac function observed in rats treated with RIPC-exosome was significantly blunted by miR-126a-3p antagomir treatment as evidenced by increased the ΔLVEF (9.3 ± 5.1% vs. 24.2 ± 4.3%, *p* < 0.05, Figures 3J–L). Secondly, reduced myocardial infarct size in RIPC-exosome treated rats was eliminated by miR-126a-3p antagomir (31.8 ± 3.9% vs. 54.4 ± 9.7%, *p* < 0.05, Figure 3M). Third, reduced myocardial apoptosis in RIPC-exosome treatment was shown the same change (35.5 ± 5.1 vs. 51.3 ± 5.1%, *p* < 0.05, Figure 3N).

In sum, these results suggest that miR-126a-3p was an important component in cardioprotection induced by plasma exosomes at the late phase of RIPC.

### miR-126a-3p Was an Important Molecule in Cardioprotection

**Figure 4:**
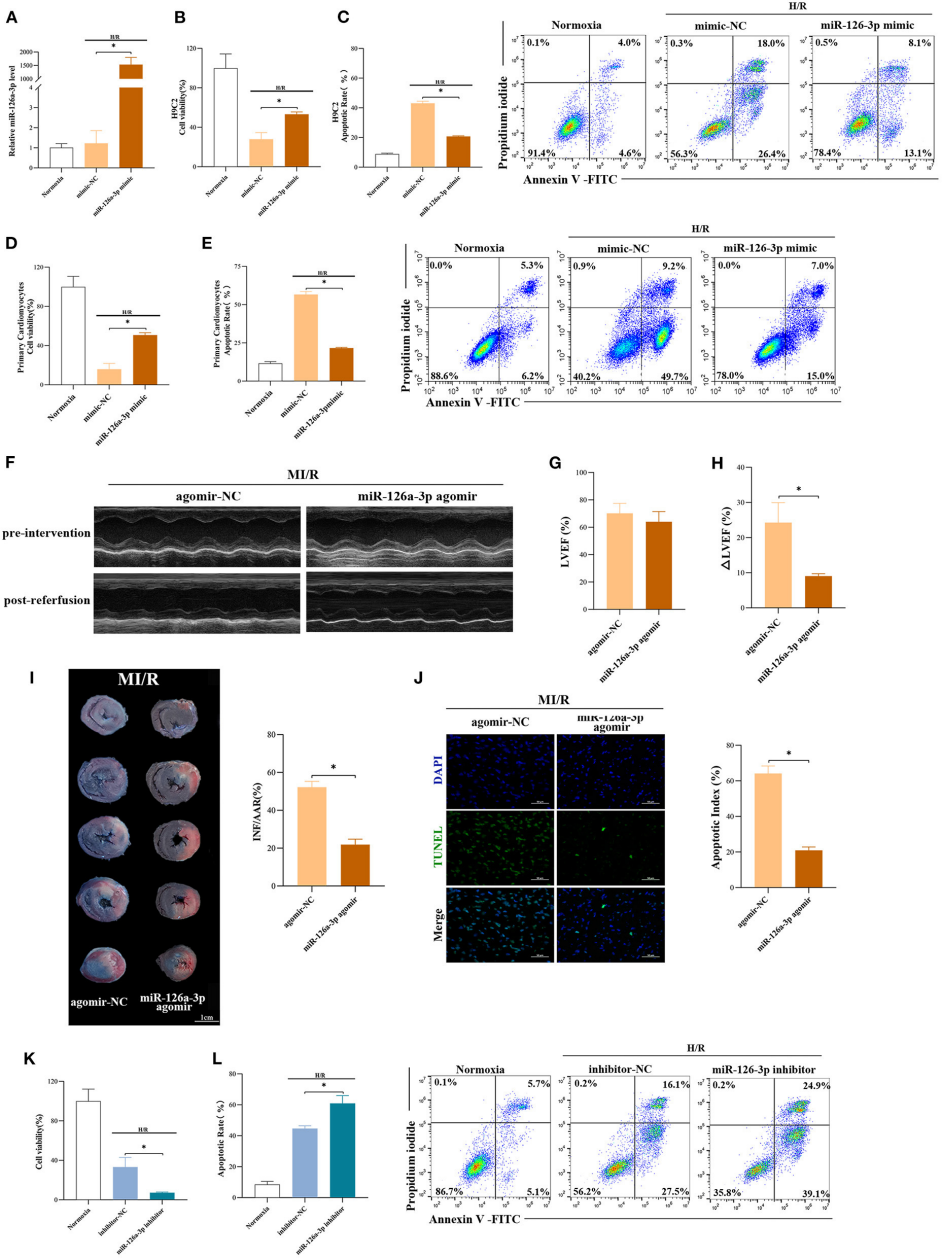
miR-126a-3p was an important molecule in cardioprotection. **(A)** The qRT-PCR analysis of miR-126a-3p level in H9C2 cells transfected with miR-126a-3p mimic or negative control (mimic-NC) for 24 h followed by H/R. Data were presented as a fold change of the normoxia. U6 was used as an internal control (*n* = 3). **(B)** The increasing level of miR-126a-3p improved cell viability in H9C2 cells subjected to H/R (*n* = 3). **(C)** Flow cytometric data and representative images of apoptosis in H9C2 cells subjected to H/R (*n* = 3). The increasing level of miR-126a-3p decreased cell apoptosis in H9C2 cells subjected to H/R. **(D)** Cell viability in primary cardiomyocytes transfected with a miR-126a-3p mimic or mimic-NC followed by H/R injury (*n* =3). **(E)** Flow cytometric data and representative images of the anti-apoptosis effect of miR-126a-3p in primary cardiomyocytes treated with a miR-126a-3p mimic or mimic-NC followed by H/R injury (*n* =3). **(F)** Representative echocardiographic images of rats in pre-intervention and post-reperfusion in each group of rats (*n* = 5). **(G)** Left ventricular ejection fraction (LVEF) of rats in pre-intervention. **(H)** ΔLVEF in each group showed that miR-126a-3p agomir improved cardiac function. **(I)** Representative photographs of heart sections and quantified data demonstrated that miR-126a-3p agomir reduced myocardial infarct size (*n* = 5). **(J)** Representative images and quantified data of TUNEL assay in each group demonstrated that miR-126a-3p agomir reduced myocardial apoptosis (*n* = 4). Scale bar: 50 μm. **(K)** Cell viability in H9C2 cells treated with a miR-126a-3p inhibitor or inhibitor-NC followed by H/R (*n* = 3). **(L)** Flow cytometric data and representative images of apoptosis in H9C2 cells treated with a miR-126a-3p inhibitor or inhibitor-NC followed by H/R (*n* = 3). H/R, Hypoxia/Reoxygenation (24/6 h). MI/R, myocardial ischemia/reperfusion (30/180 min). *p < 0.05.

To further explore the function of miR-126a-3p, miR-126a-3p mimic was transfected to increase miR-126a-3p expression in cells and then established the model of H/R (Supplementary Figure 5A). It showed that miR-126a-3p with increased expression level significantly improved cell viability and reduced cell apoptosis in both H/R induced H9C2 cells and primary cardiomyocytes (Figures 4A–E). Besides, *in vivo*, miR-126a-3p agomir was injected to detect the direct effect of miR-126a-3p against MI/R injury in rats (Supplementary Figure 5B). The cardiac function (9.1 ± 0.7% vs. 24.3 ± 5.7%, *p* < 0.05, Figures 4F–H), the myocardial infarct size (21.9 ± 2.9% vs. 52.2 ± 3.1%, *p* < 0.05, Figure 4I) and the myocardial apoptosis (21.0 ± 1.9% vs. 64.2 ± 4.2%, *p* < 0.05, Figure 4J) were significantly improved, compared with the miR-126a-3p agomir group and the agomir-NC group.

Meanwhile, to better understand the function of miR-126a-3p, we additionally performed experiments using miR-126a-3p inhibitors in H9C2 cells (Supplementary Figure 5C). Following H/R injury, decreased cell viability and increased cell apoptosis were showed in H9C2 cells transfected with miR-126a-3p inhibitor (Figures 4K,L).

Collectively, these results suggested that miR-126a-3p might be a possible and important molecule of some of the cardioprotection against MI/R injury.

### The Signaling Pathways Involved in Plasma Exosomes at the Late Phase of RIPC Mediated Effects

**Figure 5:**
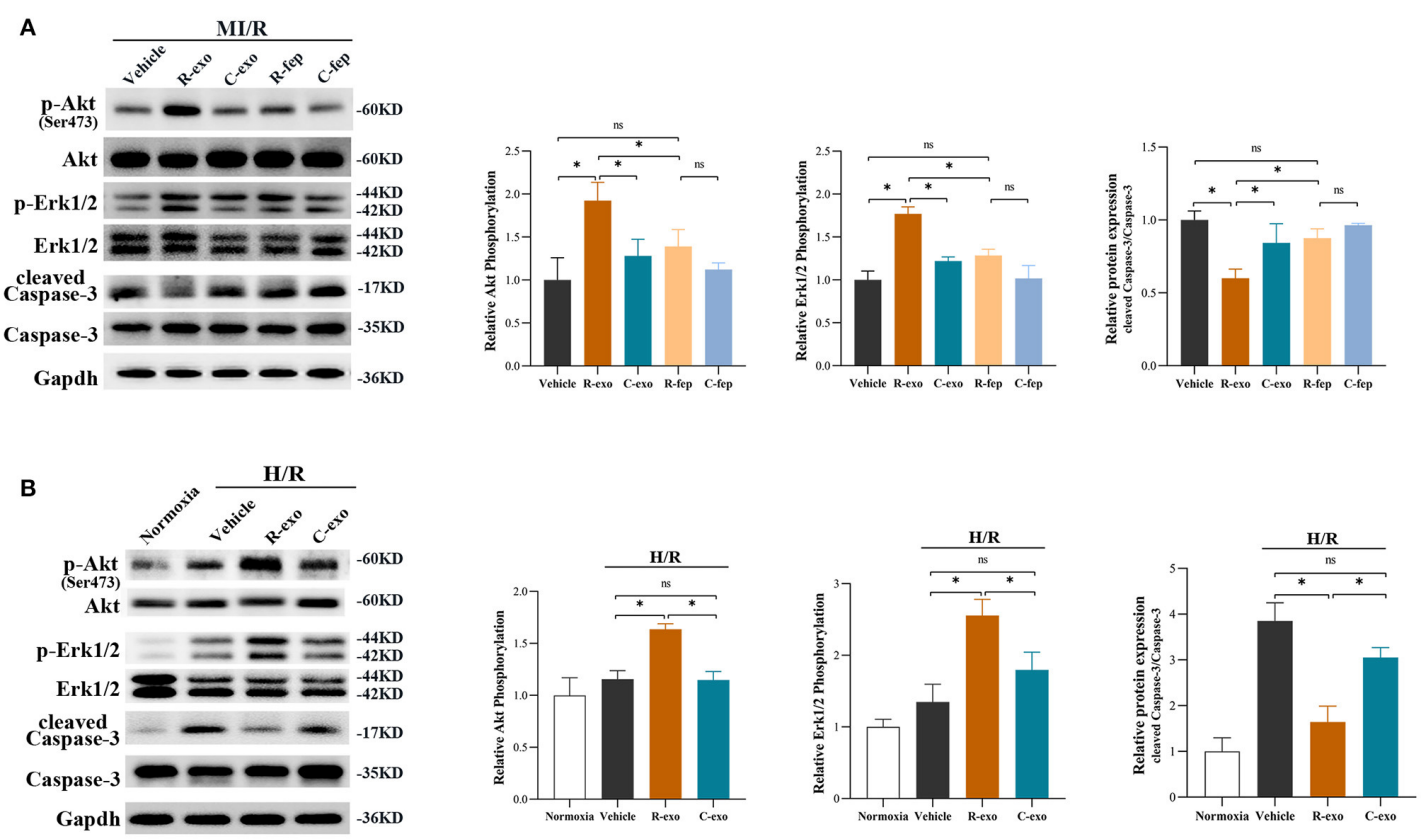
The signaling pathways involved in plasma exosomes at the late phase of RIPC mediated effects. **(A)** Representative images of Western blot and quantified data in rats received R-exo, C-exo, R-fep, C-fep, or vehicle for 24 h followed by MI/R. (*n* = 3). **(B)** Representative images of Western blot and quantified data in H9C2 cells preincubated with R-exo, C-exo, or vehicle for 24 h followed by H/R (*n* = 3). GAPDH was used as a loading control. Quantified data showed phosphorylation level of Akt, expressed as the ratio of p-Akt to Akt; phosphorylation level of Erk1/2, expressed as the ratio of p-Erk1/2 to Erk1/2; the activation of Caspase-3 expressed as the ratio of cleaved Caspase-3 to Caspase-3. MI/R, myocardial ischemia/reperfusion (30/180 min). H/R, Hypoxia/Reoxygenation (24/6 h). R-exo, exosomes isolated from the plasma of the late-phase RIPC rats; C-exo, exosomes isolated from the plasma of the control rats; R-fep, the supernatant collected after the first ultracentrifugation from the plasma of the late-phase RIPC rats; C-fep, the supernatant collected after the first ultracentrifugation from the plasma of the control rats. *p < 0.05, ^ns^*p* > 0.05.

Survival signals Akt and Erk1/2, which belong to the reperfusion injury salvage kinase (RISK) pathway, have been well-documented as key molecules in cardioprotection (35, 36). RIPC-exosome (R-exo) significantly increased the phosphorylation of Akt and Erk1/2 compared to Control-exosome or free-exosome plasma in rats subjected to MI/R injury (Figure 5A). The phosphorylation of Akt and Erk1/2 in the R-exo group was the most obvious and significantly different from that in the other groups, respectively. *In vitro*, similar results were observed in H9C2 cells subjected to H/R injury (Figure 5B). We also explored the Akt and Erk1/2 intervened by pathway inhibitors (LY294002 or U0126) in RIPC-exosome (Supplementary Figures 6A,B).

Moreover, we focused on Caspase-3 which is involved in both MI/R injury and the apoptosis pathway (Figure 5A). We found that RIPC-exosome significantly decreased the activation of Caspase-3 (cleaved Caspase-3/Caspase-3) compared to Control-exosome or free-exosome plasma in rats subjected to MI/R injury (Figure 5A). *In vitro*, similar results were observed in H9C2 cells subjected to H/R injury (Figure 5B).

Taken together, these results showed that RIPC-exosome exerted regulation over Akt and Erk1/2 activities and inhibited the activation of apoptotic protein Caspase-3.

### The Signaling Pathways Involved in Plasma Exosomes at the Late Phase of RIPC via miR-126-3p Mediated Effects

**Figure 6:**
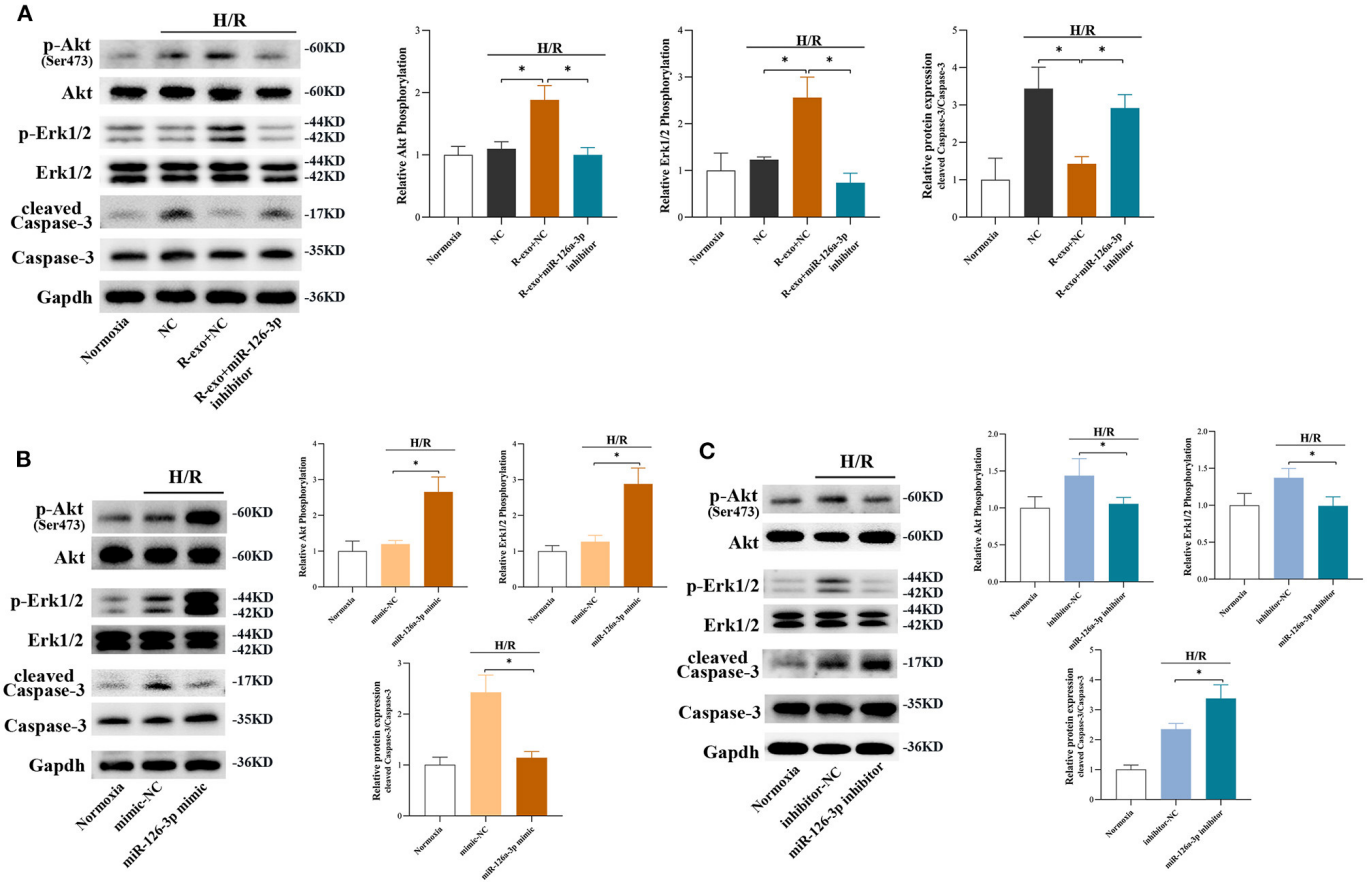
The signaling pathways involved in plasma exosomes at the late phase of RIPC via miR-126-3p mediated effects. **(A)** Representative images of Western blot and quantified data in H9C2 cells treated with inhibitor-NC, R-exo + NC, or R-exo + miR-126a-3p inhibitor followed by H/R (*n* = 3). **(B)** Representative images of Western blot and quantified data in H9C2 cells transfected with miR-126a-3p mimics or mimic-NC for 24 h followed by H/R (*n* = 3). **(C)** Representative images of Western blot and quantified data in H9C2 cells transfected with miR-126a-3p inhibitors or negative control (inhibitor-NC) for 24 h followed by H/R (*n* = 3). GAPDH was used as a loading control. Quantified data showed phosphorylation level of Akt, expressed as the ratio of p-Akt to Akt; phosphorylation level of Erk1/2, expressed as the ratio of p-Erk1/2 to Erk1/2; the activation of Caspase-3 expressed as the ratio of cleaved Caspase-3 to Caspase-3. H/R, Hypoxia/Reoxygenation (24/6 h). R-exo, exosomes isolated from the plasma of the late-phase RIPC rats. *p < 0.05.

In H9C2 cells subjected to H/R, the phosphorylation of Akt and Erk1/2 were significantly elevated with RIPC-exosome and this change was significantly blunted by the inhibition of miR-126a-3p (Figure 6A). Besides, the activation of Caspase-3 inhibited by RIPC-exosome was significantly reversed by the inhibition of miR-126a-3p (Figure 6A). Similar anti-apoptotic effects of exosomal miR-126a-3p, namely the inhibition of Caspase-3 activation, also appeared in primary cardiomyocytes (Supplementary Figure 7A). These results showed that plasma exosomes at the late phase of RIPC activated the RISK pathway and inhibited the activation of apoptotic protein Caspase-3 through exosomal miR-126a-3p.

Moreover, in H9C2 cells subjected to H/R, the phosphorylation of Akt and Erk1/2 were elevated by a miR-126a-3p mimic (Figure 6B) and decreased by a miR-126a-3p inhibitor (Figure 6C). Simultaneously, the miR-126a-3p mimic significantly inhibited the activation of apoptotic protein Caspase-3 (Figure 6B), while the miR-126a-3p inhibitor promoted it (Figure 6C). Additionally, in primary cardiomyocytes, the activation of apoptotic protein Caspase-3 was also inhibited by a miR-126a-3p mimic (Supplementary Figure 7B), which might provide a better understanding of the anti-apoptotic effect of miR-126a-3p.

Collectively, the results indicated that exosomal miR-126a-3p exerted regulation over Akt and Erk1/2 activities and inhibited the activation of apoptotic protein Caspase-3. These effects were eliminated by transfection with miR-126a-3p inhibitor and augmented by transfection with miR-126a-3p mimic.

## Discussion

**Figure 7:**
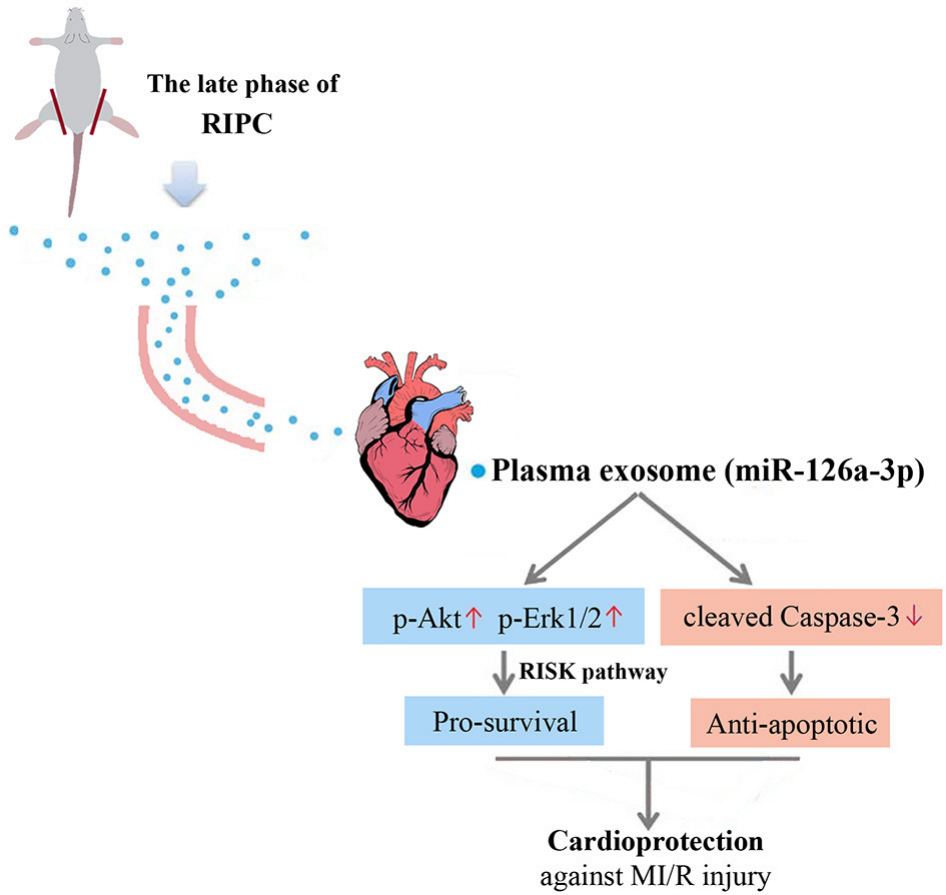
Proposed mechanisms by which plasma exosomes at the late phase of RIPC protect the heart against MI/R injury through transferring miR-126a-3p. Plasma exosomes could be used as a carrier to transfer the myocardial protective effect of RIPC at the late phase against MI/R injury, activate the RISK pathway by increasing the phosphorylation level of Akt, and Erk1/2, and inhibit the activation of apoptotic protein Caspase-3 by reducing the expression level of cleaved Caspase-3.

This study yielded three major findings. First, plasma exosomes obtained at the late phase of RIPC (RIPC-exosome) had protective effects against MI/R injury, suggesting that plasma exosomes were a carrier of protective substances induced by RIPC at the late phase. Second, exosomal miR-126a-3p played an important role in the cardioprotection induced by RIPC-exosome. Third, RIPC-exosome conferred cardioprotection through transferring miR-126a-3p by activating the RISK pathway and inhibiting the activation of apoptotic protein Caspase-3. These findings presented a novel mechanism for the endogenous cardioprotective effects conferred by RIPC-exosome through transferring miR-126a-3p (Figure 7, Supplementary Figure 1).

Our previous studies confirmed that the cardioprotective component is present in late-phase RIPC plasma (14–17), and no specific chemical inhibitors or knockout models exist to rigorously exclude exosomes in RIPC, so we separated the exosomes from the plasma and studied the differences between the plasma with or without exosomes. Results of this study further confirmed that exosomes isolated from the late-phase RIPC plasma were important in mediating the cardioprotective effects. The MI/R injury alleviated by the RIPC-exosome relied on improving cardiac function, reducing myocardial infarction and cell apoptosis. At the same time, the RIPC-exosome also exerted vital protection against H/R injury by improving cell viability and reducing cell apoptosis when they were internalized by cardiomyocytes. There are a few studies that have proposed that exosomes induced by the RIPC at the early phase may act as carriers of the cardioprotective factors (25, 26). Vincencio et al. determined that that endogenous plasma exosomes from rats and humans subjected to RIPC were powerfully cardioprotective in tested models of MI/R injury. Mechanically, they found the whole process of myocardial protection not only depended on toll-like receptor 4, further activation of Erk1/2, but also MAPK signaling pathways, following phosphorylation of the HSP27 (25). Similarly, Wen et al. verified rats subjected to RIPC produced a higher expression of miR-24 in plasma exosomes. More importantly, miR-24 contributed to improving cardiomyocyte apoptosis, infarct size, and cardiac function (26). Our results, which were concentrated in the late phase of RIPC, were consistent with the above findings. Importantly, our studies have indicated that the cardioprotection of RIPC at the late phase sustains long, which means that RIPC at the late phase may be a good preventive treatment strategy and convenient to translate into clinics. Our *in vivo* and *in vitro* results revealed that the cardioprotection produced by RIPC at the late phase may translate to others through exosomes as a certain carrier, which may provide a therapeutic strategy against MI/R injury in the future. Despite these scientific conclusions, further research needs to be conducted to explore the signaling and effector mechanism of exosomes.

Increasing evidence has shown that miRNAs are related to cardiac function, disease, and cardioprotection (37–40). Regarding cardioprotection, the expression of several miRNAs, such as miR-1, miR-21, and miRNA-125b, have been identified to be significantly changed due to ischemic pre- or post-conditioning (41–44). Similarly, Li et al. suggested that miR-144 may be a circulating mediator of RIPC and a potential biomarker for the successful application of pre-conditioning (45). However, to date, the expression profiles of miRNAs in RIPC-exosome and their potential roles in RIPC-related cardioprotection against MI/R injury are still unknown. In this study, we thus determined the expression profile of miRNAs in RIPC-exosome. We found 23 miRNAs with higher expression in RIPC-exosome, of which miR-126a-3p has the highest expression. Recent studies have revealed that miR-126a-3p plays an important role in prohibiting apoptosis and promoting the survival of endothelial cells, promoting angiogenesis and secretion of angiogenic factors (46–50). The reduced miR-126 expression has also been identified as a novel mechanism that limits cardiac functional improvement (51, 52). Circulating miR-126 has been found to play a role in cardiac repair during cardiovascular injury (21, 22). Therefore, we were focused on miR-126a-3p in this study and tested its potential involvement in cardioprotection induced by RIPC-exosome. However, other miRNAs which were aberrantly expressed in RIPC-exosome, are also significant enough for investigation in future studies.

We hypothesized that miR-126a-3p might be a critical participant in cardioprotection induced by RIPC-exosome. To test it, we first determined the function of exosomal miR-126a-3p. The result demonstrated that the cardioprotective effect of RIPC-exosome was significantly eliminated by miR-126a-3p inhibitor transfection *in vitro* and miR-126a-3p antagomir treatment *in vivo*. To further test our hypothesis, miR-126a-3p mimic was applied in cultured H9C2 cells before they were damaged by H/R injury. We found that H/R-induced injuries on H9C2 cells were attenuated via miR-126a-3p overexpression. In addition, the opposite results were showed in H9C2 cells transfected with miR-126a-3p inhibitor. The study by Wang et al. demonstrated that miR-126 may play a protective role in MI/R injury through regulating ERRFI1 (53). Wang et al. differed from us in that they focused on the role of miR-126 in MI/R injury, but did not study the role of exosomes. Our data supplied the first glimpse of the role of plasma exosomes at the late phase of RIPC against MI/R injury, and attempted to condense the protective effect of RIPC into exosomes and exosomal miR-126a-3p, so that RIPC might play a protective role in a more convenient, efficient, and lasting form, providing new ideas for the prevention and treatment of MI/R injury.

Recently, exosomes and miRNAs have been considered as potential biomarkers of heart injury and potential therapeutic tools for cardiovascular disease (54–56). Exosomes have been proposed to be involved in the range of cardiovascular processes and show great potential for discovery and application of new therapeutic strategies, as well as for diagnosis. Exosomes have several advantages over existing methods. They are biocompatible, stable, non-immunogenic, non-tumorigenic, and freeze-thaw resistant. Also, they can circulate throughout the body and cross the blood-brain barrier (57, 58). According to the existing research results, researchers believe that miRNAs have a good prospect for clinical transformation, and some miRNAs are currently being studied for clinical transformation. For example, miR-92a, miR-208, and miR-15 in the treatment of heart failure, hypertensive cardiomyopathy, and myocardial infarction are undergoing preclinical studies (59); and miR-29b mimic (MRG201-30-001) in the treatment of pathological fibrosis is entering phase I clinical study; miR-122 antagonist (SPC3649) for hepatitis C has entered phase II clinical study (60). Current exosome purification and identification techniques limit the use of exosomes as biomarkers for cardiovascular disease. Although some researchers have proposed to use exosomes as drug delivery carriers, the safety, quality control and pharmacokinetics of exosomes need to be comprehensively evaluated before implementation (61, 62). In our study, the myocardial protective effect of exosomal miR-126a-3p was confirmed by the models of MI/R injury *in vivo* and H/R injury *in vitro*, providing a new idea for the treatment methods and therapeutic targets for the cardioprotection against myocardial ischemia/reperfusion injury.

The reperfusion injury salvage kinase (RISK) pathway which consists of several survival protein kinases including Akt and Erk1/2, has been considered to confer powerful myocardial protection induced by ischemic pre-conditioning (63–66). Previous studies have shown that the activation of survival signaling Akt and Erk1/2 pathways and apoptosis-signaling pathway inactivation may represent an important mechanism in the cardioprotection conferred by RIPC (67–69). And our previous studies suggested that the RISK pathway might be involved in the cardioprotection of preconditioned plasma at the late phase of RIPC (17). Therefore, our study continued to explore the phosphorylation levels of Akt and Erk to verify whether the RISK pathway was involved in myocardial protection induced by the plasma exosomes at the late phase of RIPC through miR-126a-3p. Our present *in vivo* and *in vitro* studies both showed that, under the intervention of RIPC-exosome, the phosphorylation of Akt, Erk1/2 were significantly increased, but cleaved Caspase-3 was significantly decreased. Further study showed that these differential expressions induced by RIPC-exosome were inhibited by the miR-126a-3p inhibitor. It also showed that the phosphorylation of Akt, Erk1/2 increased and cleaved Caspase-3 decreased in cardiomyocytes preincubated with the miR-126a-3p mimic. The opposite results were showed in cardiomyocytes preincubated with the miR-126a-3p inhibitor. These further confirm the cardioprotective effects of the RIPC plasma exosomal miR-126a-3p, affected by enhancing Akt and Erk1/2 signaling and exerting anti-apoptotic effects by targeting Caspase-3 in cardiomyocytes.

Previous studies have shown that early-phase RIPC leads to an increased number of plasma exosomes (25, 26). However, this study showed that late-phase RIPC did not significantly increase the number of exosomes in plasma, but significantly altered the contents especially the level of the miRNA in exosomes. This result was interesting and may suggest a different mechanism between early- and late-phase RIPC. The exact reasons are not clear. It may be that the increase in the number of exosomes released at the early phase of RIPC is transient, and it will be adjusted by itself after some time.

This study has some limitations. First, there is currently a lack of animal models or specific inhibitors for blocking exosome production *in vivo*, which precludes *in vivo* loss-of-function studies with RIPC-induced exosomes concerning cardioprotection. Second, although the results demonstrated the protective role of plasma exosomes at the late phase of RIPC, the free-exosome plasma at the late phase of RIPC also had the potential for cardioprotection. This result suggested that there may be also other protective materials in pre-conditioned plasma, although exosomes were more representative and applicable. Third, the protective mechanism of plasma exosomes at the early phase and the late phase may be different. Further study was needed to explore the reason for the differences between plasma exosomes at the early phase and the late phase. Fourth, we focused more on the cardioprotective effect of plasma exosomes at the late phase of RIPC and only made a preliminary exploration on the mechanism.

## Conclusion

In conclusion, the late-phase RIPC derived plasma exosomes may be a carrier to transfer the cardioprotection against myocardial ischemia-reperfusion injury and exosomal miR-126a-3p was identified as an important cardioprotective molecule, which activated the RISK pathway and inhibited the activation of apoptotic protein Caspase-3. These findings present a novel mechanism underlying the exosomes at the late phase RIPC transferred cardioprotection against MI/R injury through miR-126a-3p. Exosomal miR-126a-3p might be a novel cardioprotective molecule in the prevention and rehabilitation of MI/R injury.

## Data Availability Statement

The original contributions presented in the study are publicly available. This data can be found here: GEO accession number: GSE182675.

## Ethics Statement

The animal study was reviewed and approved by Animal Care Ethics Committees of the Sixth Affiliated Hospital, Sun Yat-sen University.

## Author Contributions

SJ supervised the study and revised the manuscript. DL performed the experiments, analyzed data, and drafted the manuscript. YZha designed the study and revised the manuscript. CZ and FW performed the experiments. YZho analyzed the data. All authors contributed to the article and approved the submitted version.

## Funding

This research was supported by grants from the National Natural Science Foundation of China (Grant No. 81600396).

## Conflict of Interest

The authors declare that the research was conducted in the absence of any commercial or financial relationships that could be construed as a potential conflict of interest.

## Publisher's Note

All claims expressed in this article are solely those of the authors and do not necessarily represent those of their affiliated organizations, or those of the publisher, the editors and the reviewers. Any product that may be evaluated in this article, or claim that may be made by its manufacturer, is not guaranteed or endorsed by the publisher.

## Supplementary Material

Supplementary Figure 1 Schematic diagram of the experimental design. Plasma exosomes, isolated from the plasma of the rats subjected to the late phase of RIPC, exert cardioprotection against MI/R injury. By miRNA sequencing and subsequent functional validation, the late-phase RIPC-induced exosomal miR-126a-3p was identified as an important cardioprotective molecule. Exosomes and miR-126a-3p exerted cardioprotective effects against MI/R injury through enhancing the survival signaling (p-Akt and p-Erk1/2) in the RISK pathway and inhibiting the activation of apoptotic protein Caspase-3 in the apoptotic pathway. RIPC, remote ischemic pre-conditioning; MI/R, myocardial ischemia/reperfusion; H/R, hypoxia/ reoxygenation.

Supplementary Figure 2 Flowchart of study design for the effect of exosomes. **(A)** *In vitro* Study design. H9C2 cells were assigned to 4 groups (*n* = 3). The normoxia group included cells in a normal culture without H/R injury. The vehicle group included cells subjected to H/R. The R-exo group included cells pre-incubated with RIPC-exosome 24 h before H/R injury. The C-exo group included cells pre-incubated with Control-exosome 24 h before H/R injury. **(B)** *In vivo* study design. Forty rats were randomized into 5 groups including the vehicle group, R-exo group, C-exo group, R-fep group, and C-fep group (*n* = 8). The rats in the vehicle group were injected with 1 ml of PBS. The rats in the R-exo group were injected with RIPC exosomes isolated from equal volumes (1 ml) of plasma. The rats in the C-exo group were injected with control exosomes isolated from equal volumes (1 ml) of plasma. The rats in the R-fep group were injected with 1 ml of free-exosome plasma from the RIPC rats. The rats in the C-fep group were injected with 1 ml of free-exosome plasma from the control rats. The fluid in each group was injected from the tail vein 24 h before the MI/R injury procedure. The experiments were performed by an investigator blinded to group allocation. Four rats in total died during the procedure. In the vehicle group, one died due to surgery. In the C-exo group, one died due to an anesthesia accident. In the C-fep group, two died of pneumothorax and surgery. H/R, Hypoxia/Reoxygenation (24/6 h). MI/R, myocardial ischemia/reperfusion (30/180 min). R-exo, exosomes isolated from the plasma of the late-phase RIPC rats; C-exo, exosomes isolated from the plasma of the control rats; R-fep, the supernatant collected after the first ultracentrifugation from the plasma of the late-phase RIPC rats; C-fep, the supernatant collected after the first ultracentrifugation from the plasma of the control rats.

Supplementary Figure 3 qRT-PCR analysis of miR-126a-3p expression in rats' myocardium via tail-vein injection in MI/R-injury. Twenty-four hours after the tail vein injection of R-exo, C-exo, or PBS, rats were subjected to MI/R, then myocardial RNAs were extracted and detected by qRT-PCR (*n* = 4). R-exo, exosomes isolated from the plasma of the late-phase RIPC rats; C-exo, exosomes isolated from the plasma of the control rats. MI/R, myocardial ischemia/reperfusion (30/180 min). Data are normalized to U6. ^*^*p* < 0.05.

Supplementary Figure 4 Flowchart of study design for the effect of miR-126a-3p in plasma exosomes at the late phase of RIPC. **(A)** The *in vitro* study design for evaluating the effects of exosomal miR-126a-3p in cells. Cells were assigned to 4 groups. Cells were assigned to be transfected with miR-126a-3p inhibitors or negative control (inhibitor-NC) for 24 h and then pre-incubated with RIPC-exosome for 24 h followed by H/R. The normoxia group included cells in a normal culture without H/R injury. The NC+H/R group included cells transfected with inhibitor-NC at 100 nmol/L and then subjected to H/R injury. The R-exo + NC + H/R group included cells pre-incubated with RIPC exosomes and transfected with inhibitor-NC at 100 nmol/L and then subjected to H/R injury. The R-exo + miR-126a-3p inhibitor + H/R group included cells pre-incubated with RIPC exosomes and transfected with miR-126a-3p inhibitor at 100 nmol/L and then subjected to H/R injury. **(B)** The *in vivo* study design for evaluating the effects of exosomal miR-126a-3p in rats. Fifteen rats were randomly assigned to 3 groups and were intracardiac injected 24 h before the MI/R (*n* = 5). The rats in the NC group were intramyocardially injected with 10 nmol/L antagomir-NC. The rats in the R-exo + NC group were intramyocardially injected with 10 nmol/L antagomir-NC and RIPC-exosome isolated from 1 ml RIPC-plasma. The rats in the R-exo + miR-126a-3p antagomir group were intramyocardially injected with 10 nmol/L miR-126a-3p antagomir and RIPC-exosome isolated from 1 ml RIPC-plasma. In the miR-126-3p antagomir group, a rat died in an anesthesia accident. MI/R, myocardial ischemia/reperfusion (30/180 min). R-exo, exosomes isolated from the plasma of the late-phase RIPC rats; C-exo, exosomes isolated from the plasma of the control rats.

Supplementary Figure 5 Flowchart of study design for the effect of miR-126a-3p. **(A)** The *in vitro* study design for evaluating the direct effects of the miR-126a-3p in cells. Cells were assigned to 3 groups (*n* = 3). Cells were assigned to be transfected with miR-126a-3p mimics or negative control (mimic-NC) for 24 h followed by H/R. The miR-126a-3p mimic and mimic-NC were used at a concentration of 50 nmol/L. The normoxia group included cells in a normal culture without H/R injury. The mimic-NC+H/R group included cells transfected with mimic-NC and then subjected to H/R injury. The miR-126a-3p mimi + H/R group included cells transfected with miR-126a-3p mimic and then subjected to H/R injury. **(B)** The *in vivo* study design for evaluating the direct effects of the miR-126a-3p in rats. Ten rats were randomly assigned to 2 groups and intracardiac injected miR-126a-3p agomir or agomir-NC 24 h before the MI/R, including the agomir-NC group and miR-126a-3p agomir group (*n* = 5). The rats in the agomir-NC group were injected with 5 nmol/L agomir-NC. The rats in the miR-126a-3p agomir group were intramyocardially injected with 5 nmol/L miR-126a-3p agomir. In the miR-126-3p agomir group, a rat died in the surgery. **(C)** The *in vitro* study design for evaluating the effects when miR-126a-3p were inhibited in H9C2 cells. H9C2 cells were assigned to 3 groups (*n* = 3). H9C2 cells were assigned to be transfected with miR-126a-3p inhibitors or negative control (inhibitor-NC) for 24 h followed by H/R. The miR-126a-3p inhibitor and inhibitor-NC were used at a concentration of 100 nmol/L. The normoxia group included cells in a normal culture without H/R injury. The inhibitor -NC+H/R group included cells transfected with inhibitor-NC and then subjected to H/R injury. The miR-126a-3p inhibitor + H/R group included cells transfected with miR-126a-3p inhibitor and then subjected to H/R injury. H/R, Hypoxia/Reoxygenation (24/6 h). MI/R, myocardial ischemia/reperfusion (30/180 min).

Supplementary Figure 6 The intervention by pathway inhibitors in plasma exosomes at the late phase of RIPC. Representative images of Western blot. H9C2 cells were pretreated with LY294002 (10 μM) for 1 h and then treated with RIPC-exosome or Control-exosome for 24 h before the H/R injury (*n* = 3). The increased phosphorylation of Akt in the RIPC-exosomes group was eliminated by LY294002. **(B)** Representative images of Western blot. H9C2 cells were pre-treated with U0126 (10 μM) for 1 h and then treated with RIPC-exosome or Control-exosome for 24 h before the H/R injury (*n* = 3). The increased phosphorylation of Erk1/2 in the RIPC-exosomes group was eliminated by U0126. GAPDH was used as a loading control. Quantified data showed phosphorylation level of Akt, expressed as the ratio of p-Akt to Akt; phosphorylation level of Erk1/2, expressed as the ratio of p-Erk1/2 to Erk1/2. H/R, Hypoxia/Reoxygenation (24/6 h). R-exo, exosomes isolated from the plasma of the late-phase RIPC rats; C-exo, exosomes isolated from the plasma of the control rats.

Supplementary Figure 7 The activation of apoptotic protein Caspase-3 via RIPC-exosomal miR-126-3p mediated effects in primary cardiomyocytes. **(A)** Representative images of Western blot showing activation of apoptotic protein Caspase-3 in primary cardiomyocytes treated with inhibitor-NC, R-exo ± NC, or R-exo ± miR-126a-3p inhibitor followed by H/R (*n* = 3). **(B)** Representative images of Western blot showing activation of apoptotic protein Caspase-3 in primary cardiomyocytes transfected with miR-126a-3p mimics or mimic-NC for 24 h followed by H/R (*n* = 3). GAPDH was used as a loading control. H/R, Hypoxia/Reoxygenation (24/6 h). R-exo, exosomes isolated from the plasma of the late-phase RIPC rats.
