# Supplementary figures and images for: Plasma Exosomes at the Late Phase of Remote Ischemic Pre-conditioning Attenuate Myocardial Ischemia-Reperfusion Injury Through Transferring miR-126a-3p
Source: Front Cardiovasc Med. 2021 Nov 30;8:736226. doi: 10.3389/fcvm.2021.736226 (PMC8669347; doi:10.3389/fcvm.2021.736226)

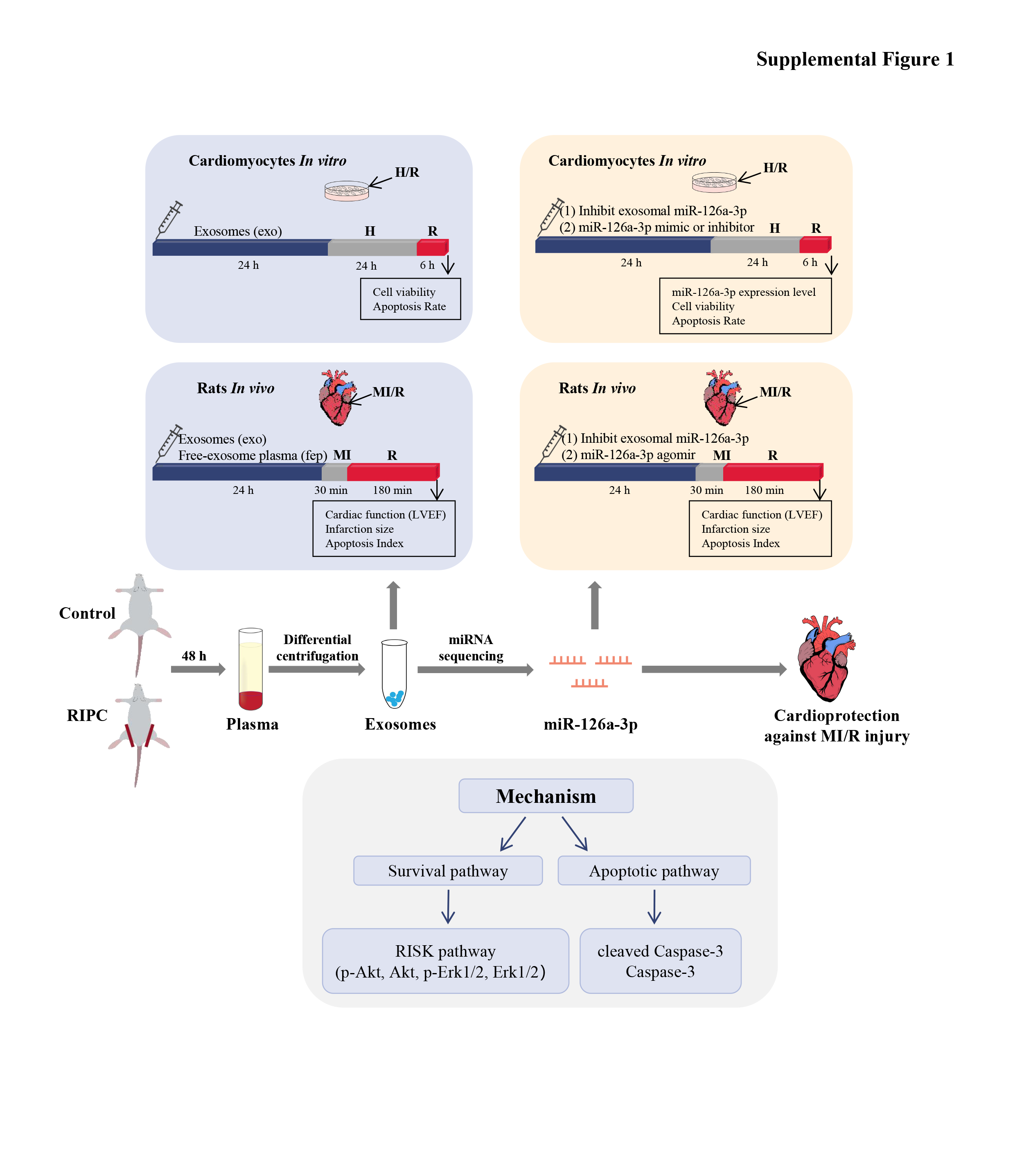

Supplement: Supplementary Figure 1 — Schematic diagram of the experimental design. Plasma exosomes, isolated from the plasma of the rats subjected to the late phase of RIPC, exert cardioprotection against MI/R injury. By miRNA sequencing and subsequent functional validation, the late-phase RIPC-induced exosomal miR-126a-3p was identified as an important cardioprotective molecule. Exosomes and miR-126a-3p exerted cardioprotective effects against MI/R injury through enhancing the survival signaling (p-Akt and p-Erk1/2) in the RISK pathway and inhibiting the activation of apoptotic protein Caspase-3 in the apoptotic pathway. RIPC, remote ischemic pre-conditioning; MI/R, myocardial ischemia/reperfusion; H/R, hypoxia/ reoxygenation. [file Image_1.tif]

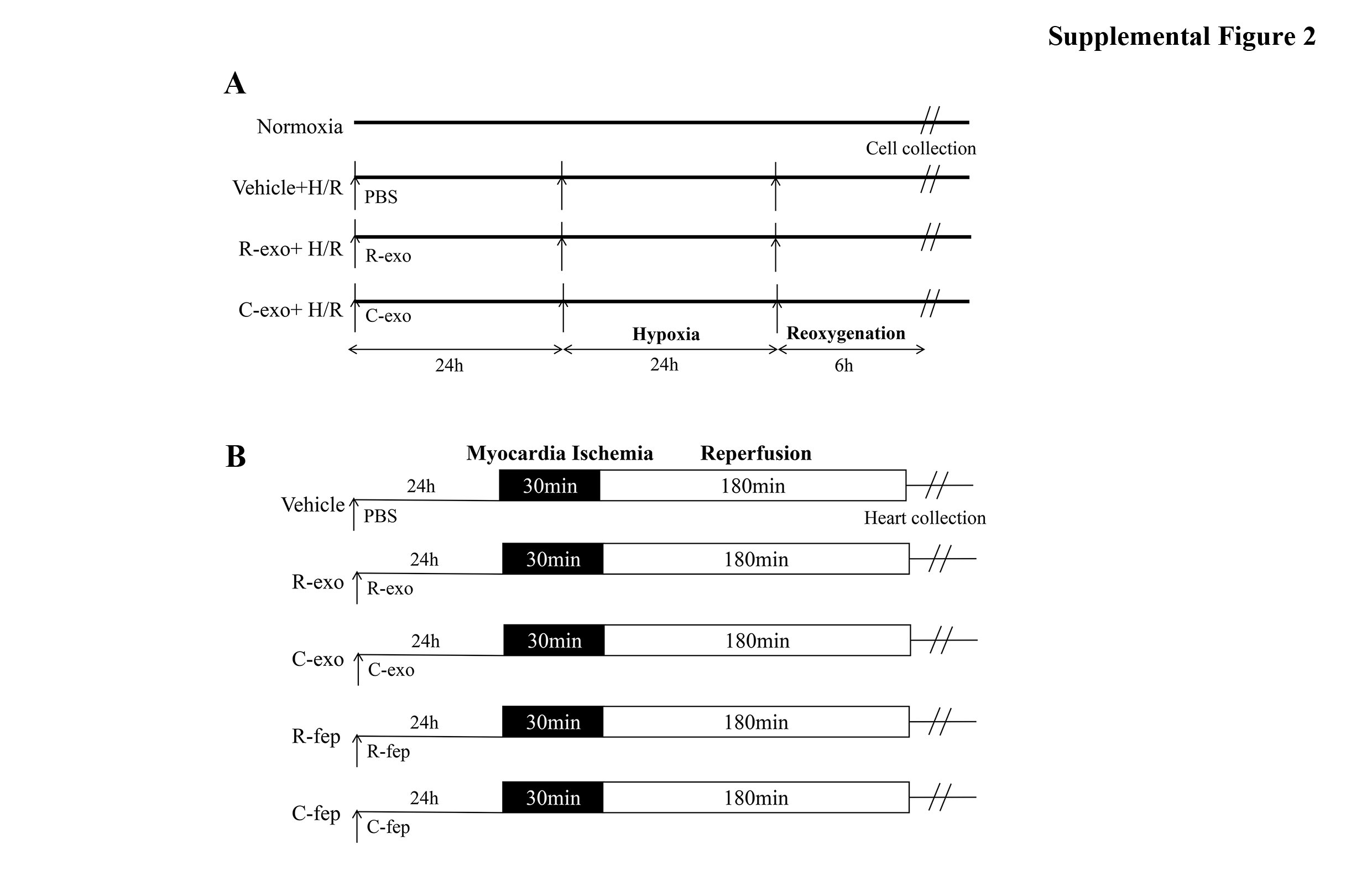

Supplement: Supplementary Figure 2 — Flowchart of study design for the effect of exosomes. (A) In vitro Study design. H9C2 cells were assigned to 4 groups (n = 3). The normoxia group included cells in a normal culture without H/R injury. The vehicle group included cells subjected to H/R. The R-exo group included cells pre-incubated with RIPC-exosome 24 h before H/R injury. The C-exo group included cells pre-incubated with Control-exosome 24 h before H/R injury. (B) In vivo study design. Forty rats were randomized into 5 groups including the vehicle group, R-exo group, C-exo group, R-fep group, and C-fep group (n = 8). The rats in the vehicle group were injected with 1 ml of PBS. The rats in the R-exo group were injected with RIPC exosomes isolated from equal volumes (1 ml) of plasma. The rats in the C-exo group were injected with control exosomes isolated from equal volumes (1 ml) of plasma. The rats in the R-fep group were injected with 1 ml of free-exosome plasma from the RIPC rats. The rats in the C-fep group were injected with 1 ml of free-exosome plasma from the control rats. The fluid in each group was injected from the tail vein 24 h before the MI/R injury procedure. The experiments were performed by an investigator blinded to group allocation. Four rats in total died during the procedure. In the vehicle group, one died due to surgery. In the C-exo group, one died due to an anesthesia accident. In the C-fep group, two died of pneumothorax and surgery. H/R, Hypoxia/Reoxygenation (24/6 h). MI/R, myocardial ischemia/reperfusion (30/180 min). R-exo, exosomes isolated from the plasma of the late-phase RIPC rats; C-exo, exosomes isolated from the plasma of the control rats; R-fep, the supernatant collected after the first ultracentrifugation from the plasma of the late-phase RIPC rats; C-fep, the supernatant collected after the first ultracentrifugation from the plasma of the control rats. [file Image_2.TIF]

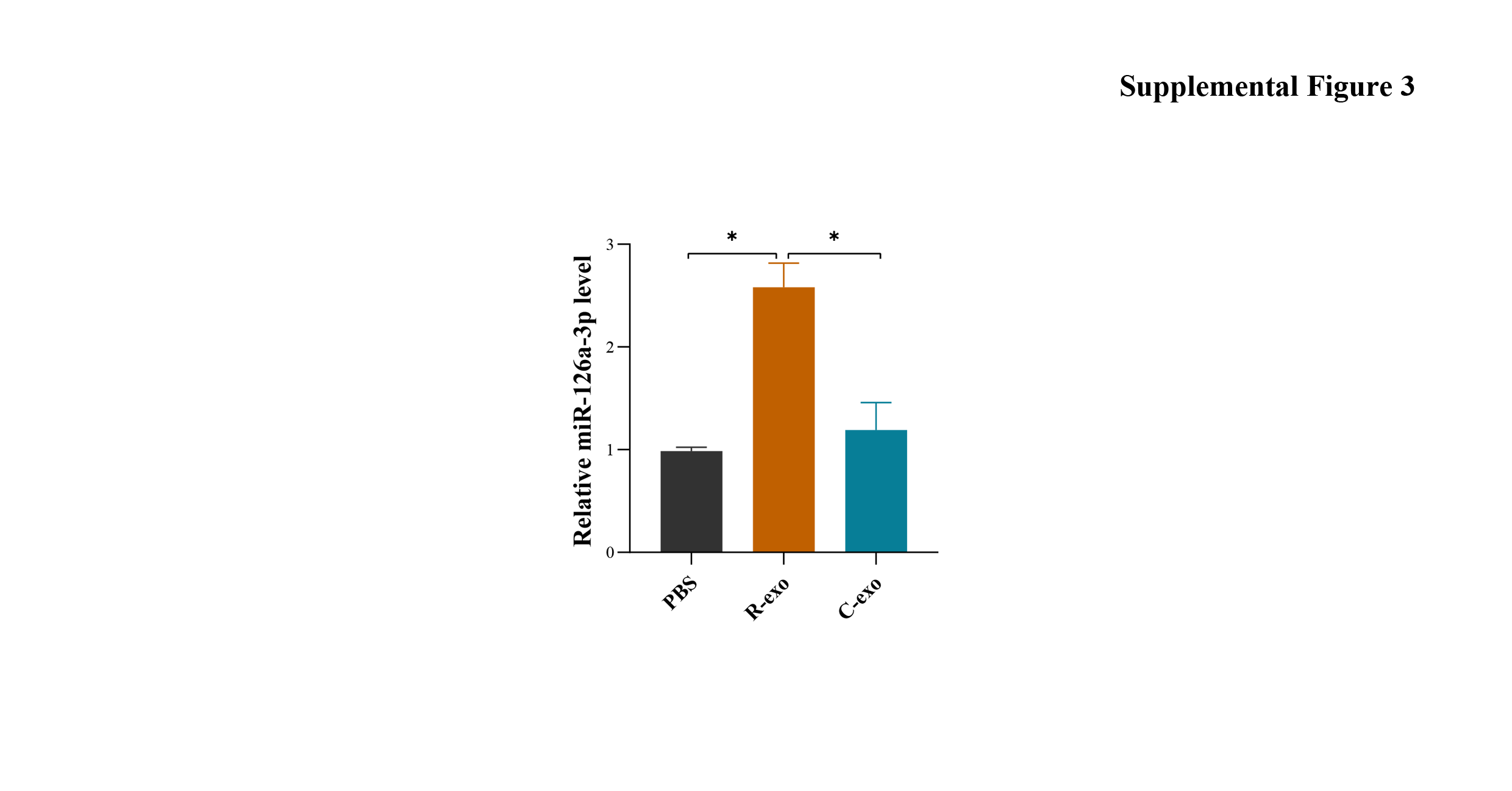

Supplement: Supplementary Figure 3 — qRT-PCR analysis of miR-126a-3p expression in rats' myocardium via tail-vein injection in MI/R-injury. Twenty-four hours after the tail vein injection of R-exo, C-exo, or PBS, rats were subjected to MI/R, then myocardial RNAs were extracted and detected by qRT-PCR (n = 4). R-exo, exosomes isolated from the plasma of the late-phase RIPC rats; C-exo, exosomes isolated from the plasma of the control rats. MI/R, myocardial ischemia/reperfusion (30/180 min). Data are normalized to U6. *p < 0.05. [file Image_3.TIF]

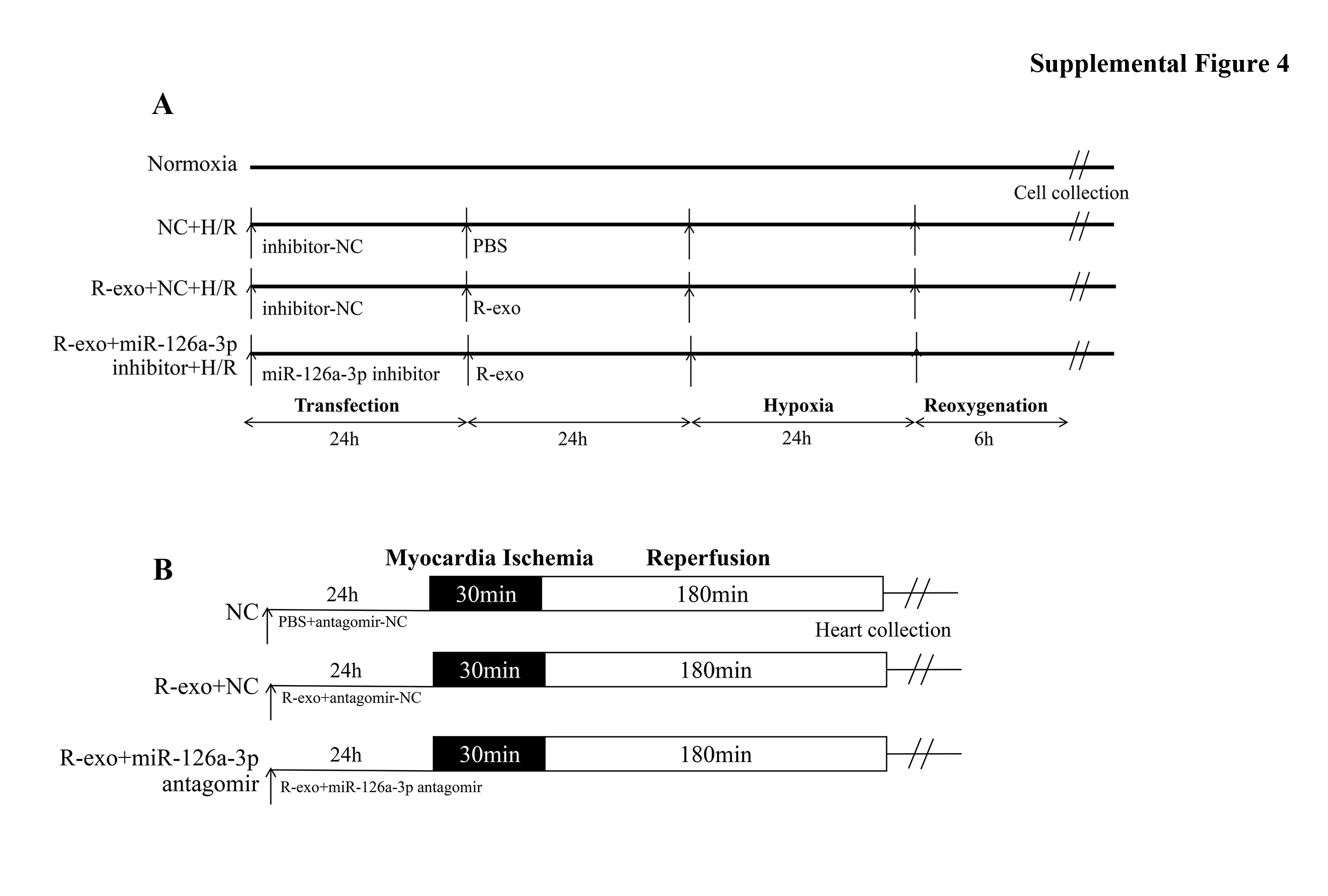

Supplement: Supplementary Figure 4 — Flowchart of study design for the effect of miR-126a-3p in plasma exosomes at the late phase of RIPC. (A) The in vitro study design for evaluating the effects of exosomal miR-126a-3p in cells. Cells were assigned to 4 groups. Cells were assigned to be transfected with miR-126a-3p inhibitors or negative control (inhibitor-NC) for 24 h and then pre-incubated with RIPC-exosome for 24 h followed by H/R. The normoxia group included cells in a normal culture without H/R injury. The NC+H/R group included cells transfected with inhibitor-NC at 100 nmol/L and then subjected to H/R injury. The R-exo + NC + H/R group included cells pre-incubated with RIPC exosomes and transfected with inhibitor-NC at 100 nmol/L and then subjected to H/R injury. The R-exo + miR-126a-3p inhibitor + H/R group included cells pre-incubated with RIPC exosomes and transfected with miR-126a-3p inhibitor at 100 nmol/L and then subjected to H/R injury. (B) The in vivo study design for evaluating the effects of exosomal miR-126a-3p in rats. Fifteen rats were randomly assigned to 3 groups and were intracardiac injected 24 h before the MI/R (n = 5). The rats in the NC group were intramyocardially injected with 10 nmol/L antagomir-NC. The rats in the R-exo + NC group were intramyocardially injected with 10 nmol/L antagomir-NC and RIPC-exosome isolated from 1 ml RIPC-plasma. The rats in the R-exo + miR-126a-3p antagomir group were intramyocardially injected with 10 nmol/L miR-126a-3p antagomir and RIPC-exosome isolated from 1 ml RIPC-plasma. In the miR-126-3p antagomir group, a rat died in an anesthesia accident. MI/R, myocardial ischemia/reperfusion (30/180 min). R-exo, exosomes isolated from the plasma of the late-phase RIPC rats; C-exo, exosomes isolated from the plasma of the control rats. [file Image_4.TIF]

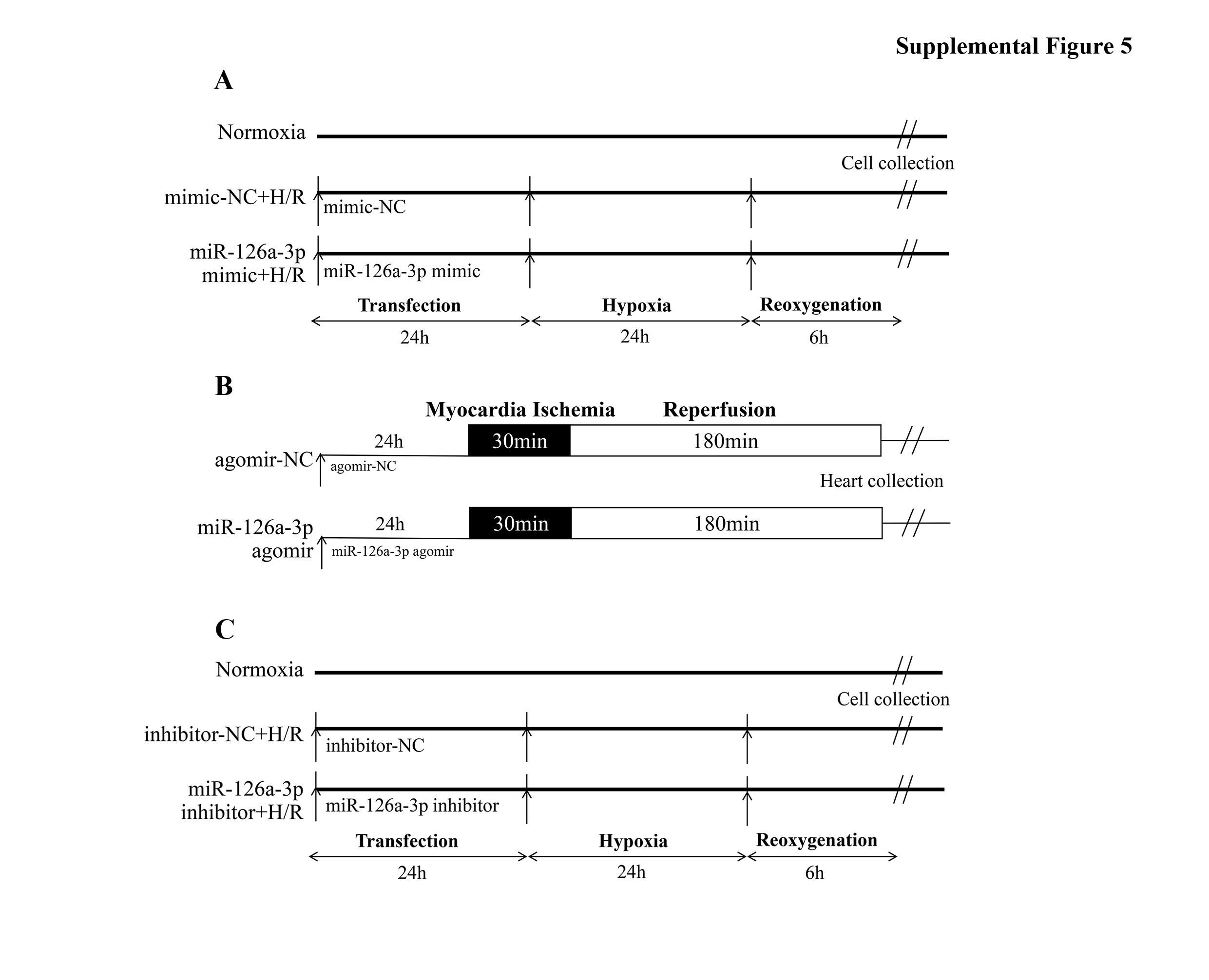

Supplement: Supplementary Figure 5 — Flowchart of study design for the effect of miR-126a-3p. (A) The in vitro study design for evaluating the direct effects of the miR-126a-3p in cells. Cells were assigned to 3 groups (n = 3). Cells were assigned to be transfected with miR-126a-3p mimics or negative control (mimic-NC) for 24 h followed by H/R. The miR-126a-3p mimic and mimic-NC were used at a concentration of 50 nmol/L. The normoxia group included cells in a normal culture without H/R injury. The mimic-NC+H/R group included cells transfected with mimic-NC and then subjected to H/R injury. The miR-126a-3p mimi + H/R group included cells transfected with miR-126a-3p mimic and then subjected to H/R injury. (B) The in vivo study design for evaluating the direct effects of the miR-126a-3p in rats. Ten rats were randomly assigned to 2 groups and intracardiac injected miR-126a-3p agomir or agomir-NC 24 h before the MI/R, including the agomir-NC group and miR-126a-3p agomir group (n = 5). The rats in the agomir-NC group were injected with 5 nmol/L agomir-NC. The rats in the miR-126a-3p agomir group were intramyocardially injected with 5 nmol/L miR-126a-3p agomir. In the miR-126-3p agomir group, a rat died in the surgery. (C) The in vitro study design for evaluating the effects when miR-126a-3p were inhibited in H9C2 cells. H9C2 cells were assigned to 3 groups (n = 3). H9C2 cells were assigned to be transfected with miR-126a-3p inhibitors or negative control (inhibitor-NC) for 24 h followed by H/R. The miR-126a-3p inhibitor and inhibitor-NC were used at a concentration of 100 nmol/L. The normoxia group included cells in a normal culture without H/R injury. The inhibitor -NC+H/R group included cells transfected with inhibitor-NC and then subjected to H/R injury. The miR-126a-3p inhibitor + H/R group included cells transfected with miR-126a-3p inhibitor and then subjected to H/R injury. H/R, Hypoxia/Reoxygenation (24/6 h). MI/R, myocardial ischemia/reperfusion (30/180 min). [file Image_5.TIF]

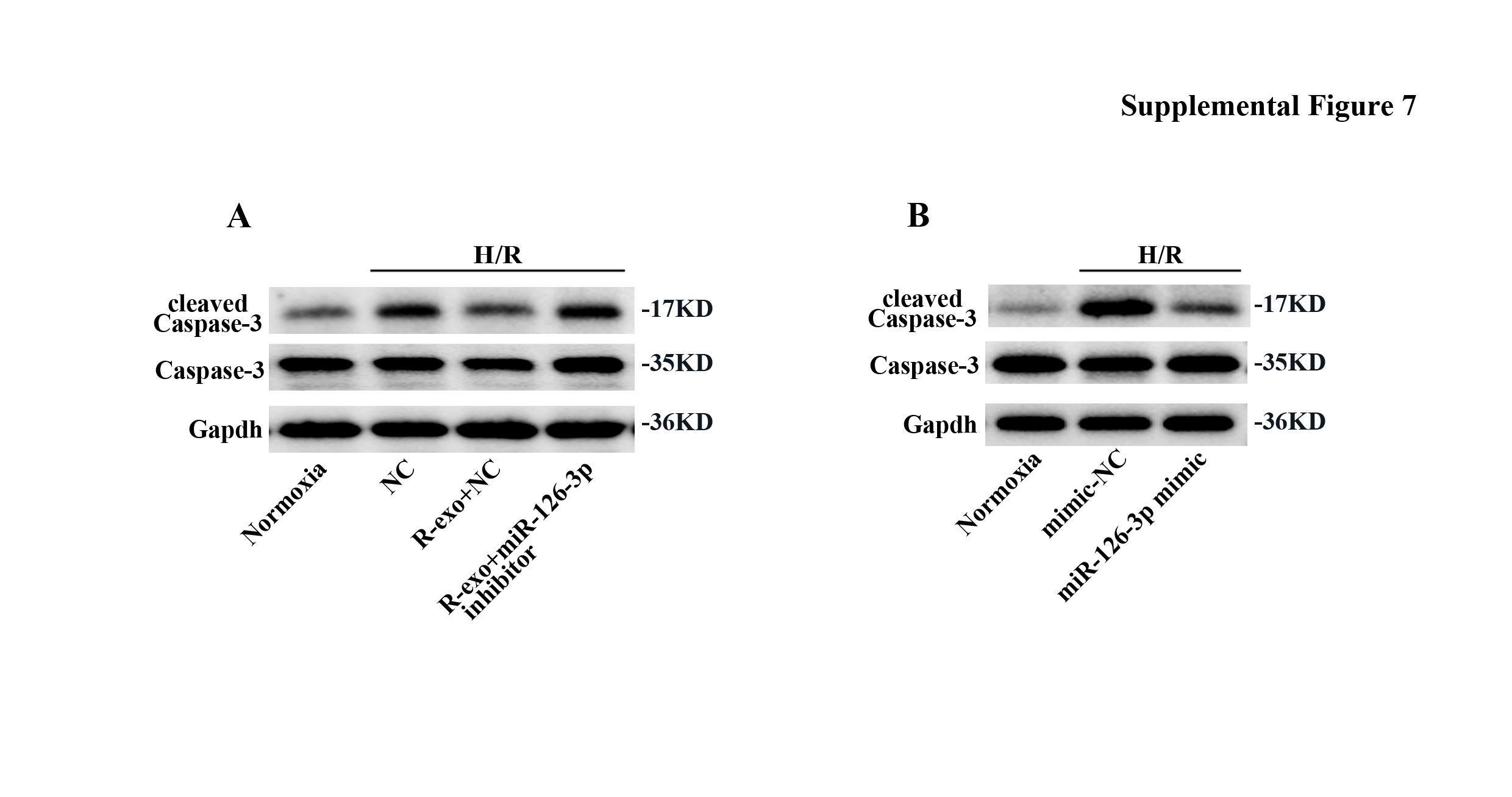

Supplement: Supplementary Figure 6 — The intervention by pathway inhibitors in plasma exosomes at the late phase of RIPC. Representative images of Western blot. H9C2 cells were pretreated with LY294002 (10 μM) for 1 h and then treated with RIPC-exosome or Control-exosome for 24 h before the H/R injury (n = 3). The increased phosphorylation of Akt in the RIPC-exosomes group was eliminated by LY294002. (B) Representative images of Western blot. H9C2 cells were pre-treated with U0126 (10 μM) for 1 h and then treated with RIPC-exosome or Control-exosome for 24 h before the H/R injury (n = 3). The increased phosphorylation of Erk1/2 in the RIPC-exosomes group was eliminated by U0126. GAPDH was used as a loading control. Quantified data showed phosphorylation level of Akt, expressed as the ratio of p-Akt to Akt; phosphorylation level of Erk1/2, expressed as the ratio of p-Erk1/2 to Erk1/2. H/R, Hypoxia/Reoxygenation (24/6 h). R-exo, exosomes isolated from the plasma of the late-phase RIPC rats; C-exo, exosomes isolated from the plasma of the control rats. [file Image_6.TIF]
